# Supplementary material for: Co-targeting JAK1/STAT6/GAS6/TAM signaling improves chemotherapy efficacy in Ewing sarcoma
Source: Nat Commun. 2024 Jun 21;15:5292. doi: 10.1038/s41467-024-49667-2 (PMC11192891; doi:10.1038/s41467-024-49667-2)
Supplement: Supplementary file 1 — Supplementary Information [file 41467_2024_49667_MOESM1_ESM.pdf]

Supplementary Information

Supplementary Fig. 1

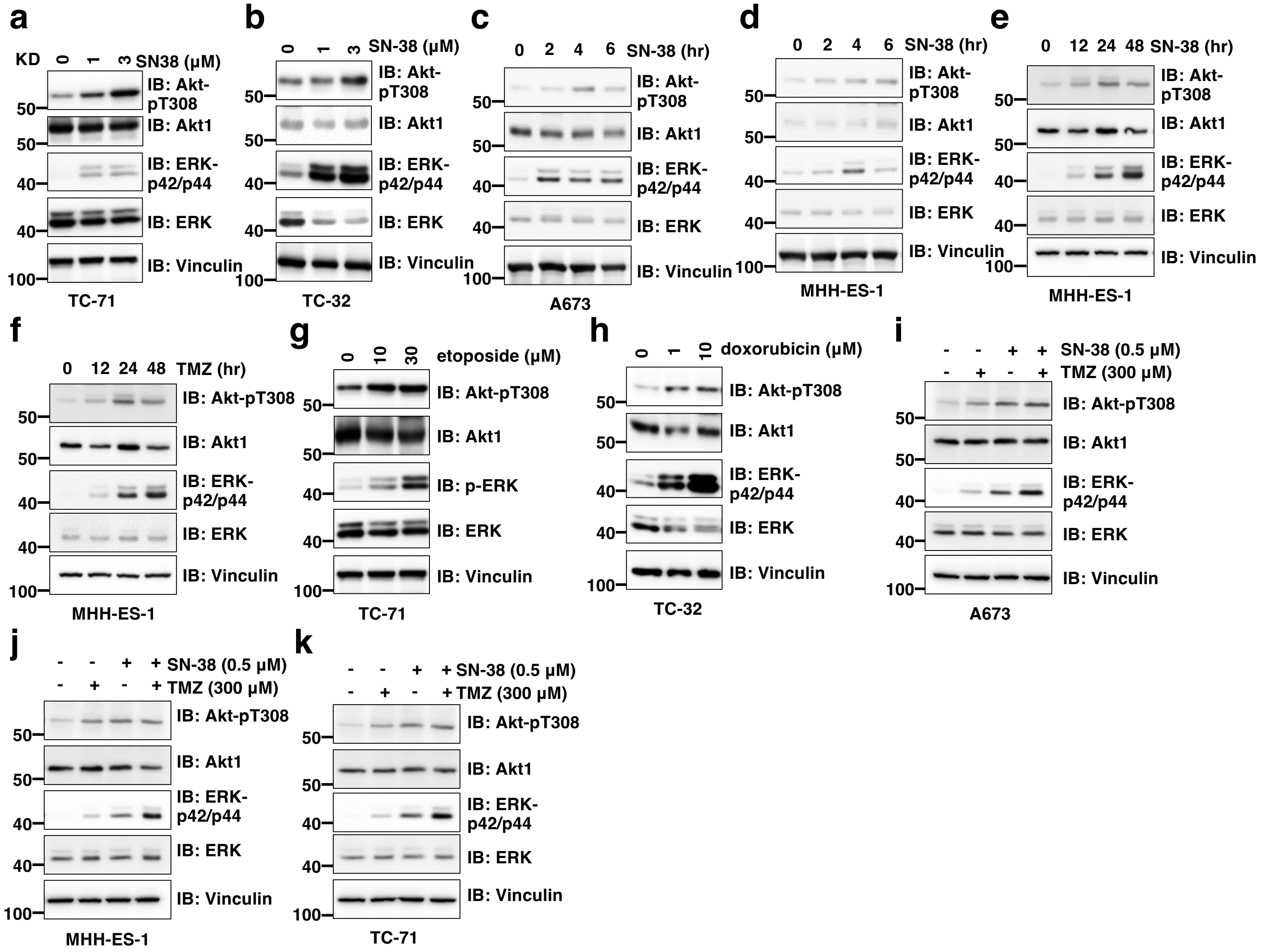

Supplementary Fig. 1 Chemotherapy induces activation of Akt and ERK in Ewing sarcoma cells. (a-b) IB analysis of WCL derived from indicated Ewing sarcoma cells treated with indicated doses chemotherapy for 24 hrs. (c-f) IB analysis of WCL derived from indicated Ewing sarcoma cells treated with indicated chemotherapy for indicated periods. (e-q) IB analysis of WCL derived from indicated Ewing sarcoma cells treated with indicated doses chemotherapy for 24 hrs. WB data presented in this figure are representative data from biological duplicates.

Supplementary Fig. 2

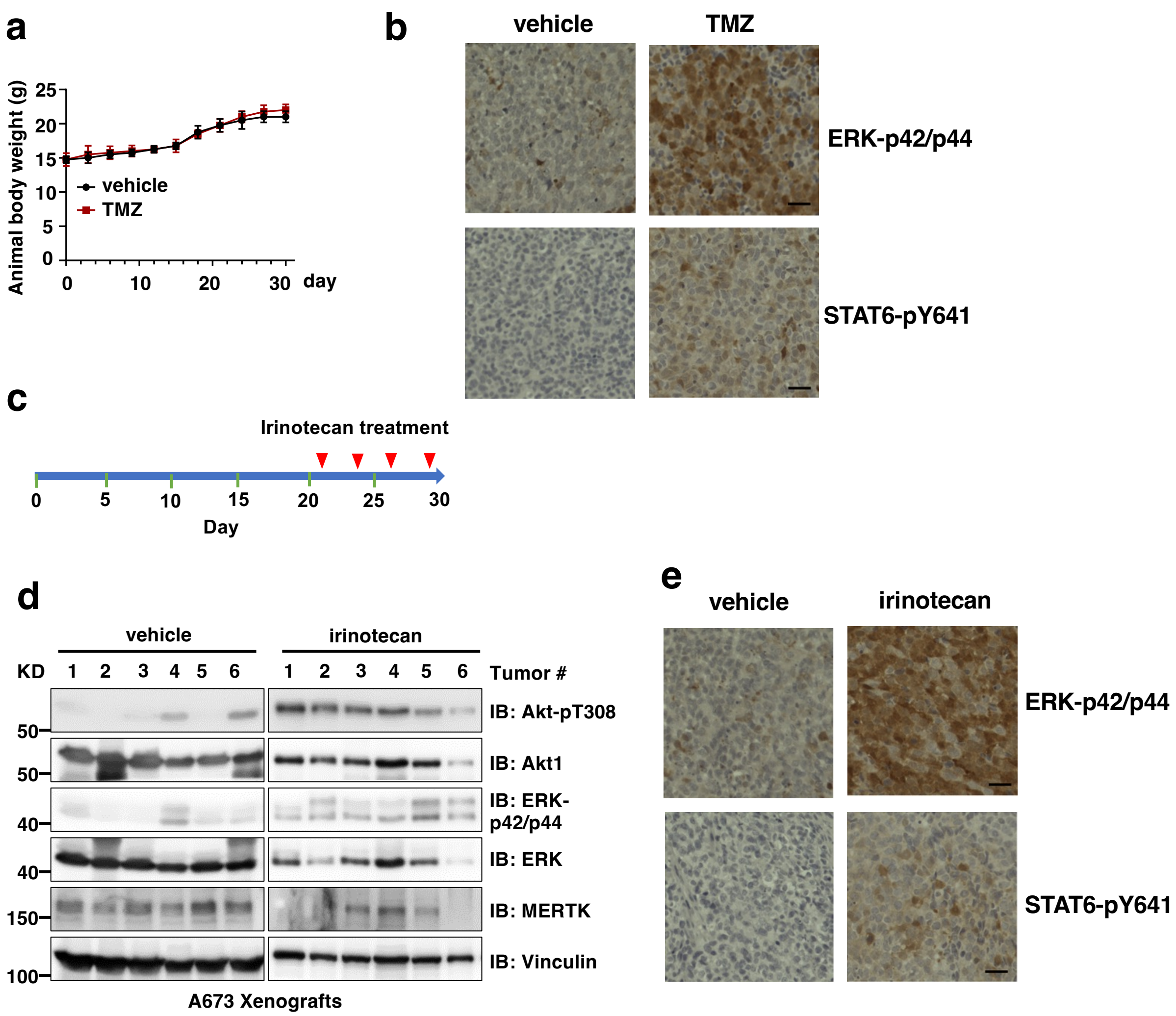

Supplementary Fig. 2 Chemotherapy induces ERK-p42/p44 signals in xenografted Ewing sarcoma tumors. (a) An animal weight measurement at indicated days post-subcutaneous injection. Error bars were calculated as mean+/-SD, n=5 animals in each group. (b) IHC analysis of collected MHH-ES-1 xenografts treated with vehicle or TMZ. The scale bar represents 100 µm. (c) A schematic schedule for irinotecan treatment starting from A673 xenografts. (d) IB analysis WCL from collected A673 xenografted tumors treated with vehicle or irinotecan. (e) Representative IHC analysis images of collected A673 xenografts treated with vehicle or irinotecan. The scale bar represents 100 µm.

Supplementary Fig. 3

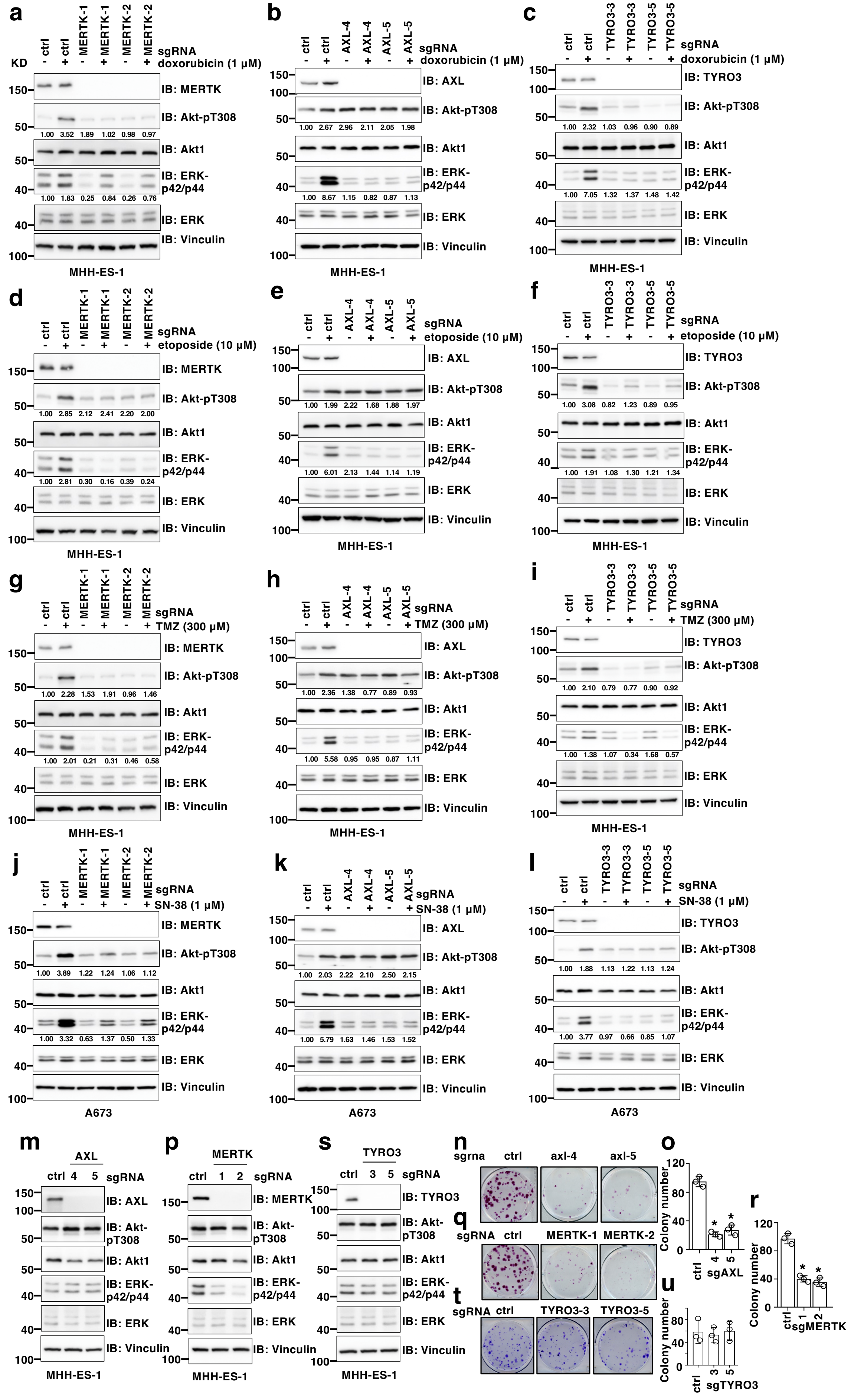

Supplementary Fig. 3 Effects of TAM kinase depletion in response to chemotherapy and genetic depletion of TAM kinase members differentially affects Ewing sarcoma cell growth *in vitro*. (a-i) IB analysis of WCL from indicated MHH-ES-1 cells treated with indicated chemotherapy for 24 hrs. (j-l) IB analysis of WCL from indicated A673 cells treated with indicated chemotherapy for 24 hrs. (m, p and s) IB analysis of WCL from MHH-ES-1 depleted of indicated TAM kinase members by sgRNAs. (n, q and t) Representative images of 2D colony formation assays using 1,000 indicated MHH-ES-1 cells for two weeks and resulting colonies are quantified on the right (o, r and u). Error bars were calculated as mean+/-SD, n=3 (biological triplicates). *p<0.05 (one-way ANOVA test). WB data presented in this figure are representative data from biological duplicates.

Supplementary Fig. 4

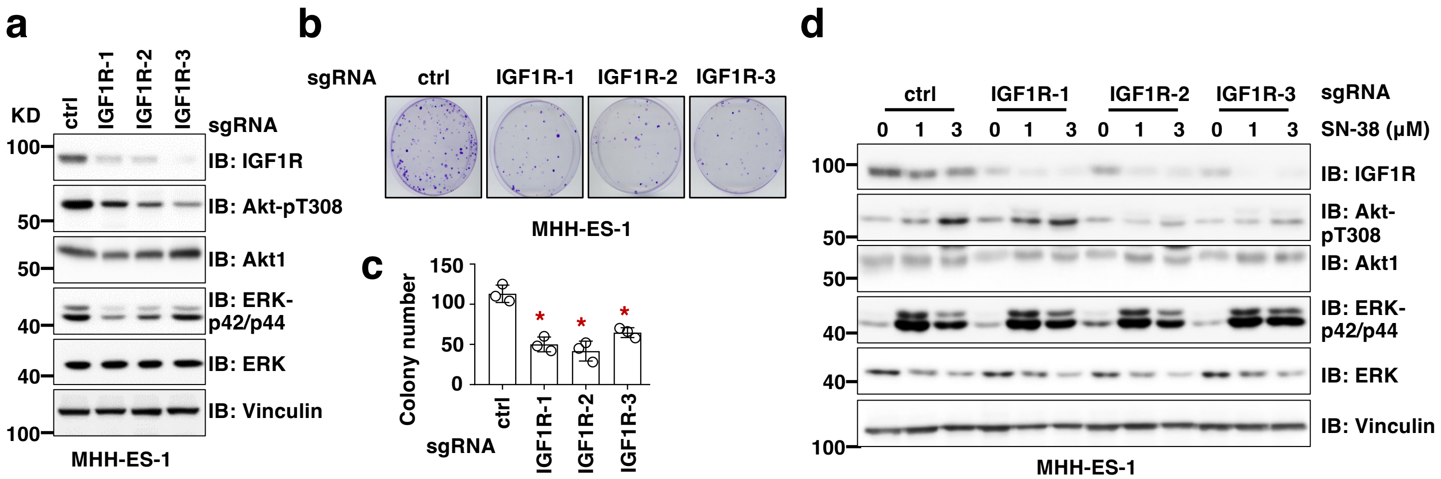

Supplementary Fig. 4 IGF1R depletion does not regulate SN-38 induced ERK activation. (a) IB analysis of control or IGF1R depleted MHH-ES-1 cells. (b) Representative images of 2D colony formation assays using 1,000 indicated MHH-ES-1 cells for two weeks and resulting colonies are quantified in (c). Error bars were calculated as mean+/-SD, n=3 (biological triplicates). *p<0.05 (one-way ANOVA test). (d) IB analysis of WCL from indicated MHH-ES-1 cells treated with indicated doses of SN-38 for 24 hrs. WB data presented in this figure are representative data from biological duplicates.

Supplementary Fig. 5

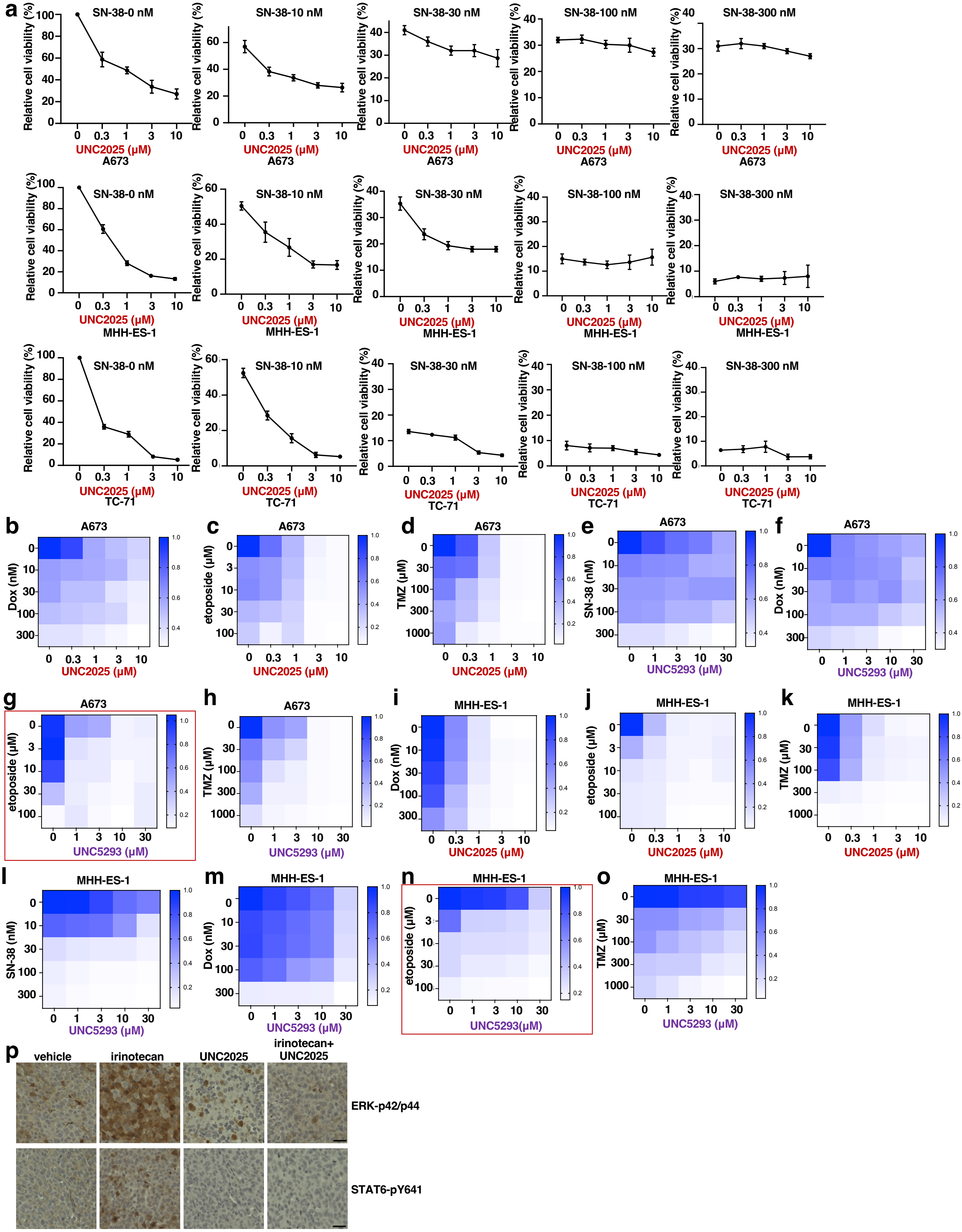

Supplementary Fig. 5 Evaluation of combinations of TAM kinase inhibitors with chemotherapeutic agents in Ewing sarcoma cell viability. (a) Representative cell viability curves for Figures 3a-3c. Error bars were calculated as mean+/-SD, n=3 (biological triplicates). (b-o) Representative heatmaps for cell viability in MHH-ES-1 cells treated with indicated doses of compounds for 2 days. (p) Representative IHC analysis images of collected MHH-ES-1 xenografts treated with vehicle, irinotecan, UNC2025 or both. The scale bar represents 100 μm.

Supplementary Fig. 6

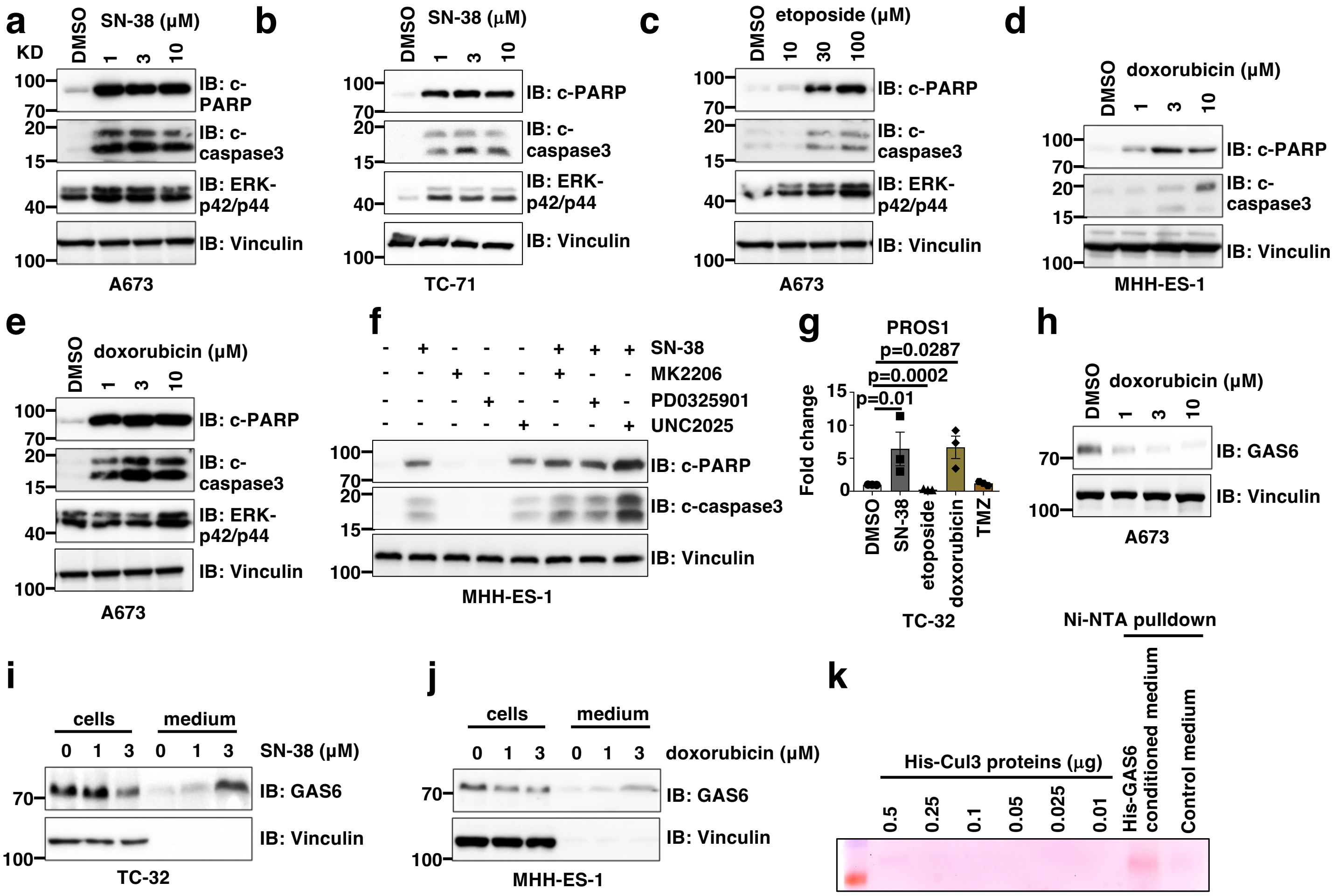

Supplementary Fig. 6 Chemotherapy induces cell apoptosis and synthesis/secretion of GAS6 in Ewing sarcoma cells. (a-e) IB analysis of indicated Ewing sarcoma cells treated with indicated chemotherapeutic agents for 24 hrs. (f) IB analysis of WCL from MHH-ES-1 cells treated with indicated compounds for 24 hrs. SN-38 (1 μM); MK2206 (100 nM); PD0325901 (100 nM); UNC2025 (1 μM). (g) RT-PCR analyses of mRNA expression changes of PROS1 in TC-32 cells treated with indicated chemotherapeutic agents for 24 hrs. SN-38, 1 μM; etoposide, 10 μM; doxorubicin, 1 μM; TMZ, 100 μM. Error bars were calculated as mean+/-SD, n=3 (biological triplicates). p values are labeled and represent differences of experimental groups compared to the control group (one-way ANOVA test). (h) IB analysis of WCL from A673 cells treated with indicated doses of doxorubicin for 24 hrs. (I, j) IB analysis of WCL and TCA-precipitated cell culture media from indicated Ewing sarcoma cells treated with indicated doses of chemotherapeutic agents for 24 hrs. (k) A ponceau S staining for Ni-NTA pulldowns from MHH-ES-1 cell culture media treated with or without 1 μM SN-38 for 24 hrs. WB data presented in this figure are representative data from biological duplicates.

Supplementary Fig. 7

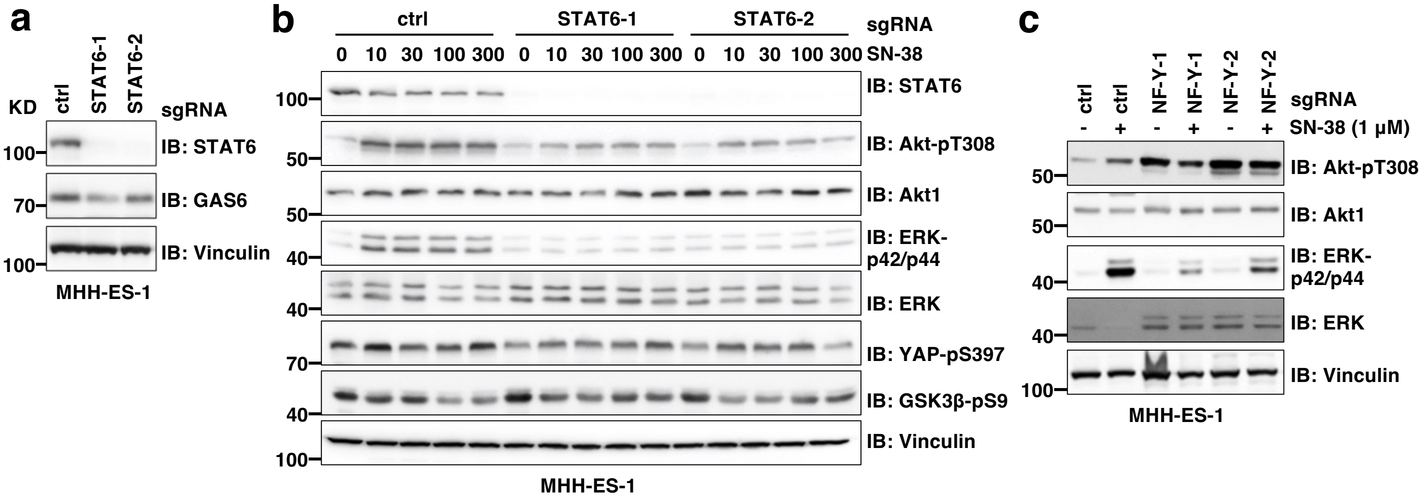

Supplementary Fig. 7 NF-Y depletion does not prevent SN-38 treatment induced Akt activation. (a) IB analysis of WCL from MHH-ES1 cells depleted of endogenous STAT6. (b) IB analysis of WCL from indicated MHH-ES-1 cells treated with indicated doses of SN-38 for 24 hrs. (c) IB analysis of WCL from MHH-ES-1 cells depleted of NF-Y and treated with 1 μM SN-38 for 24 hrs. WB data presented in this figure are representative data from biological duplicates.

Supplementary Fig. 8

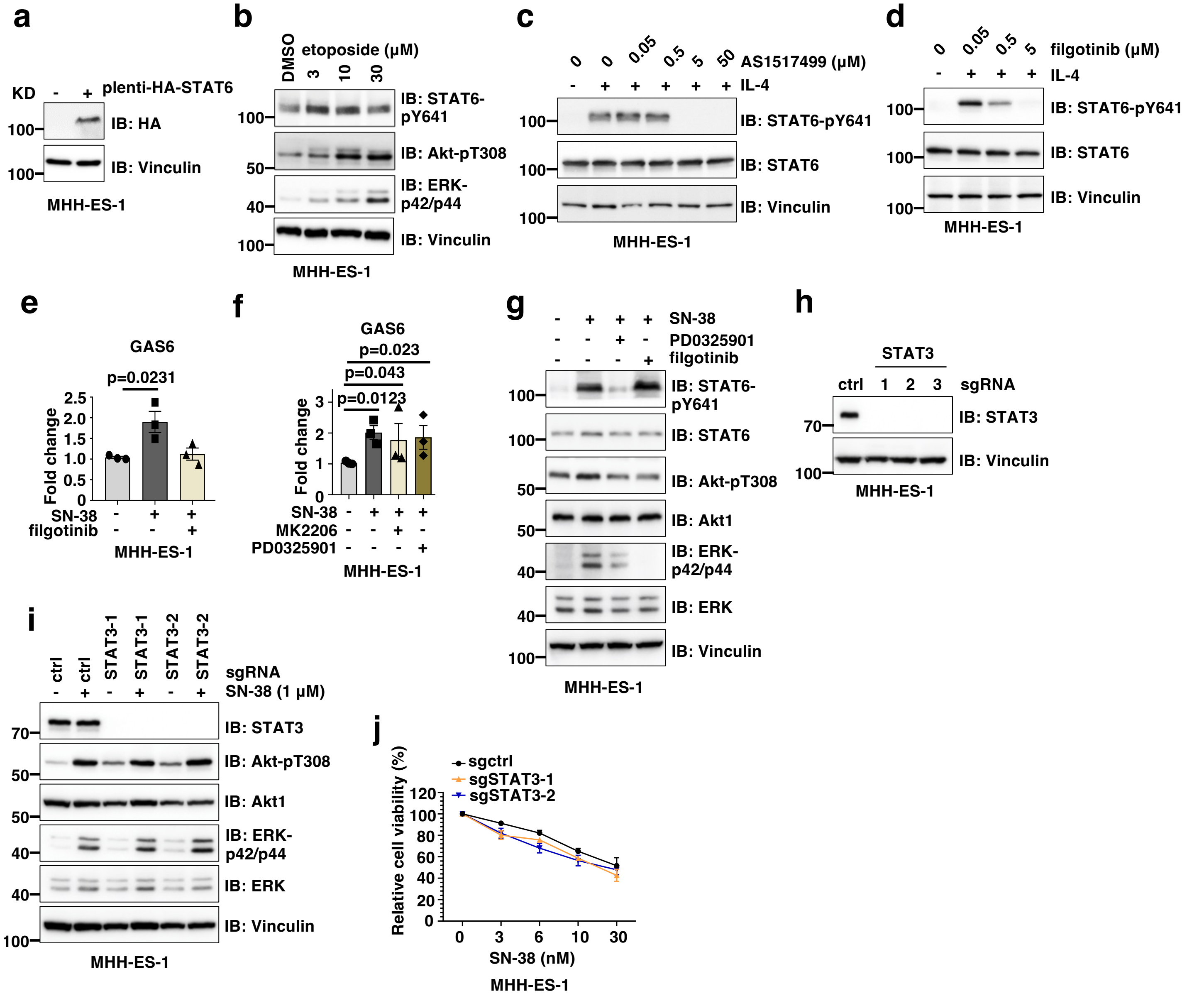

Supplementary Fig. 8 Chemotherapy induces STAT6 phosphorylation and activation. (a) IB analysis of indicated MHH-ES-1 cells. Where indicated, MHH-ES-1 cells were infected with lenti-viruses and selected with 1 μg/mL puromycin for 72 hrs to eliminate non-infected cells. (b) IB analysis of WCL from MHH-ES-1 cells treated with indicated doses of etoposide for 24 hrs. (c-d) IB analysis of WCL from MHH-ES-1 cells treated with indicated doses of AS1517499 (c) or filgotinib (d) with IL-4 for 24 hrs. (e, f) RT-PCR analyses of mRNA expression changes of GAS6 in MHH-ES-1 cells treated with indicated compounds for 24 hrs. SN-38, 1 μM; filgotinib, 5 μM; MK2206, 100 nM; PD0325901, 100 nM. Error bars were calculated as mean+/-SD, n=3 (biological triplicates). p values are labeled and represent differences of experimental groups compared to the control group (one-way ANOVA test). (g) IB analysis of WCL from MHH-ES-1 cells treated with indicated compounds for 24 hrs. SN-38, 1 μM; filgotinib, 5 μM; PD0325901, 100 nM. (h, i) IB analysis of WCL from indiated MHH-ES-1 cells treated with 1 μM SN-38 for 24 hrs. (j) Cell viability assays using control or STAT3-depleted MHH-ES-1 cells treated with indicated doses of SN-38 for 48 hrs. Error bars were calculated as mean+/-SD, n=3 (biological triplicates). WB data presented in this figure are representative data from biological duplicates.

Supplementary Fig. 9

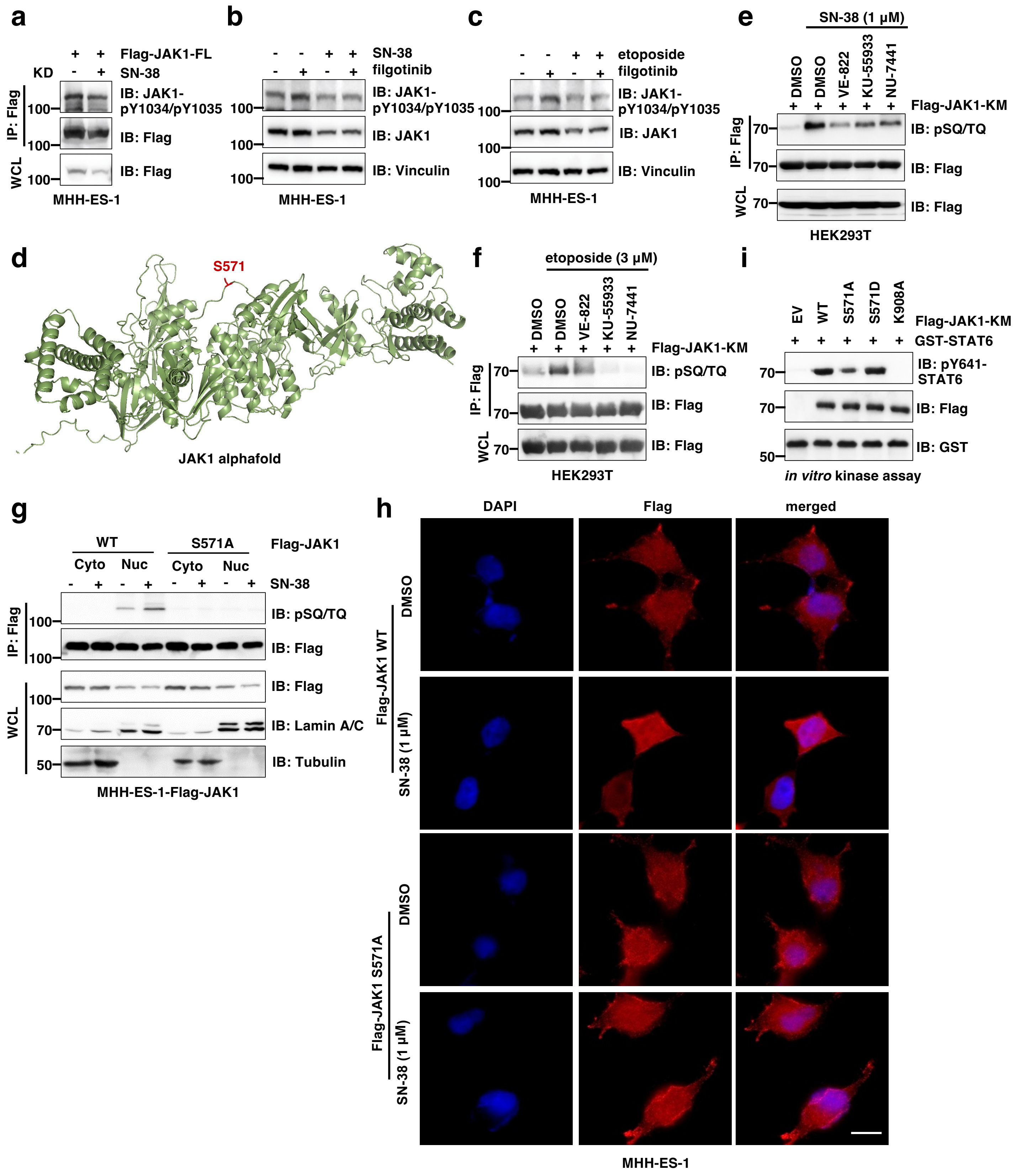

Supplementary Fig. 9 Chemotherapy induces JAK1-S571Q phosphorylation and activation. (a) IB analysis of Flag-IPs and WCL from MHH-ES-1 cells stably expressing pLenti-Flag-JAK1 treated with 1 μM SN-38 for 24 hrs. (b, c) IB analysis of WCL from MHH-ES-1 cells treated with indicated compounds for 24 hrs. SN-38, 1 μM; filgotinib, 5 μM; etoposide, 10 μM. (d) An alphafold prediction of human JAK1 structure with S571 residue labeled in red. (e, f) IB analysis of Flag-IPs and WCL from HEK293T cells transfected with Flag-JAK1-KM and treated with indicated chemotherapeutic agents with indicated kinase inhibitors for 24 hrs. VE-822, 1 μM; KU-55933, 10 μM; NU-7441, 1 μM. (g) IB analysis of Flag-IPs and WCL from MHH-ES-1 cells stably expressing indicated Flag-JAK1 treated with 1 μM SN-38 for 24 hrs and fractionated into indicated cell fractions. (h) Representative IF images for MHH-ES-1 cells expressing indicated JAK1 treated with 1 μM SN-38 for 24 hrs. The scale bar represents 20 μm. (i) *In vitro* JAK1 kinase assays using indicated immunoprecipitated Flag-JAK1-KM from HEK293T cells incubated with GST-STAT6 truncation proteins. WB data presented in this figure are representative data from biological duplicates.

Supplementary Fig. 10

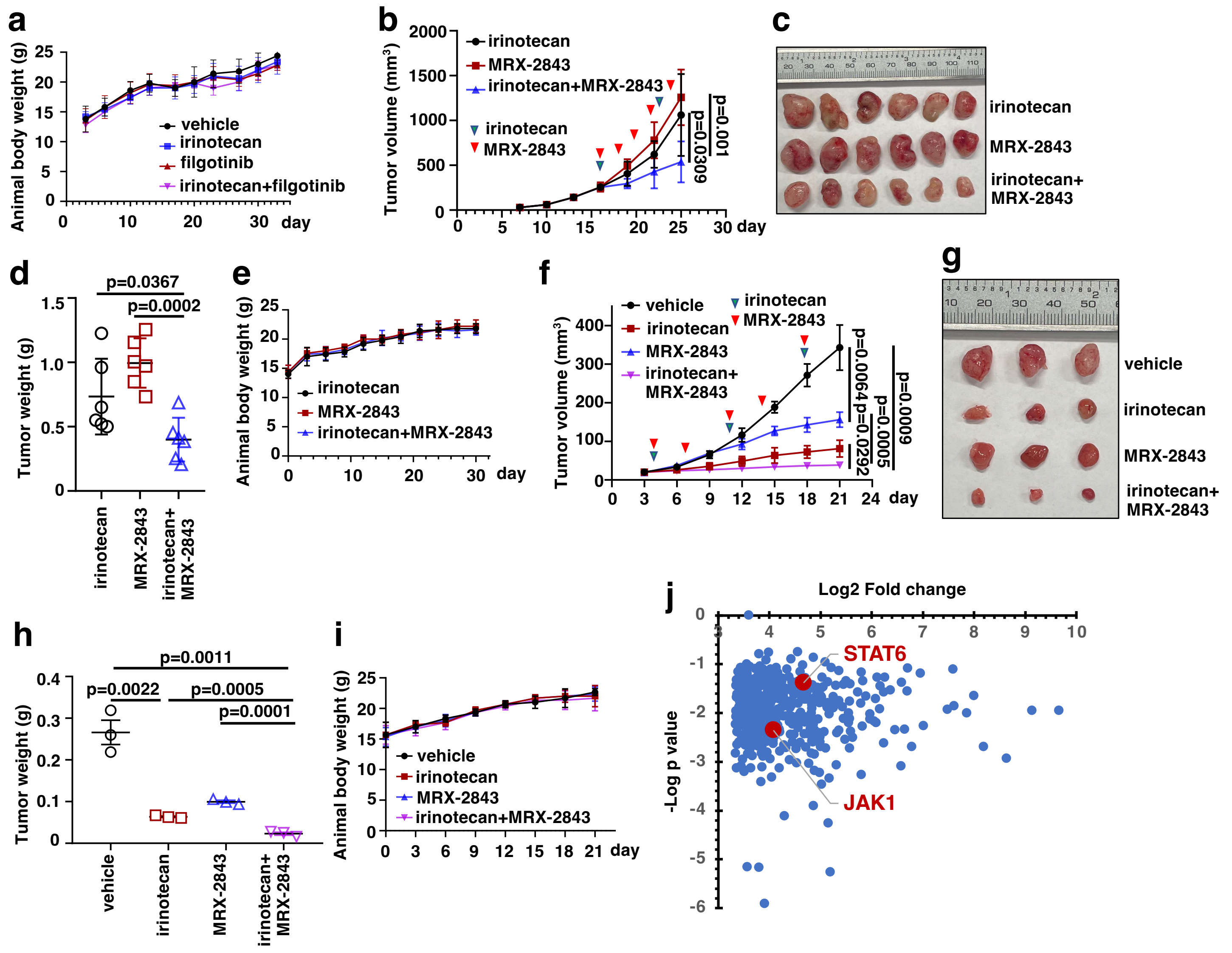

Supplementary Fig. 10 MRX-2843 enhances irinotecan effects in furhter suppressing xenografted Ewing sarcoma tumor growth. (a) The animal body weight measurements at indicated days received indicated treatments. Error bars were calculated as mean+/-SD, n=5 animals in each group. (b) A tumor volume curve for xenografted MHH-ES-1 cells treated with MRX-2843, irinotecan or both with indicated schedules. N= 6 tumors. p values are labeled and represent differences of experimental groups compared to the control group (one-way ANOVA test). (c) Dissected tumors receiving indicated treatments from b and weighed in d. Error bars were calculated as mean+/-SD, n=6 tumors. p values are labeled and represent differences of experimental groups compared to the control group (one-way ANOVA test). (e) The animal body weight measurements at indicated days received indicated treatments. Error bars were calculated as mean+/-SD, n=6 animals in each group. (f) A tumor volume curve for xenografted NCH-EWS-1 Ewing sarcoma PDX tumor cells treated with vehicle, MRX-2843, irinotecan or both with indicated schedules. Error bars were calculated as mean+/-SD, n= 3 tumors. p values are labeled and represent differences of experimental groups compared to the control group (one-way ANOVA test). (g) Dissected tumors receiving indicated treatments from e and weighed in f. n=3 tumors. (h) The animal body weight measurements at indicated days received indicated treatments. Error bars were calculated as mean+/-SD, n=3 animals in each group. p values are labeled and represent differences of experimental groups compared to the control group (one-way ANOVA test). (j) A heatmap illustrating both JAK1 and STAT6 mRNA levels are increased in Ewing sarcoma

patients poorly responding to chemotherapy compared with patients with good responses from a previous study [69].

Original uncropped images for immune blotting data in supplementary figure S1-S10

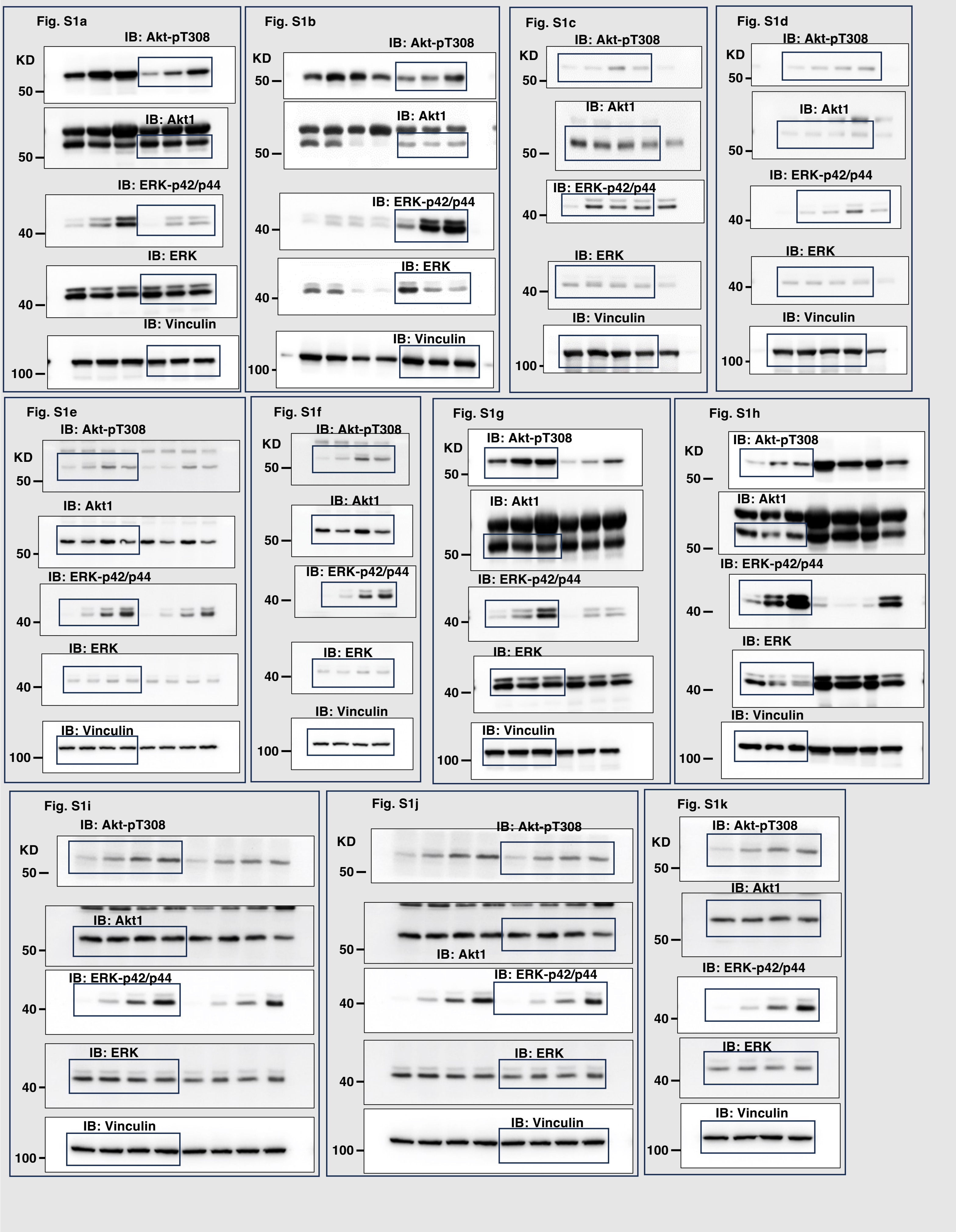
Fig. S1a
IB: Akt-pT308
KD
50
IB: Akt1
50
IB: ERK-p42/p44
40
IB: ERK
40
IB: Vinculin
100
Fig. S1b
IB: Akt-pT308
KD
50
IB: Akt1
50
IB: ERK-p42/p44
40
IB: ERK
40
IB: Vinculin
100
Fig. S1c
IB: Akt-pT308
KD
50
IB: Akt1
50
IB: ERK-p42/p44
40
IB: ERK
40
IB: Vinculin
100
Fig. S1d
IB: Akt-pT308
KD
50
IB: Akt1
50
IB: ERK-p42/p44
40
IB: ERK
40
IB: Vinculin
100
Fig. S1e
IB: Akt-pT308
KD
50
IB: Akt1
50
IB: ERK-p42/p44
40
IB: ERK
40
IB: Vinculin
100
Fig. S1f
IB: Akt-pT308
KD
50
IB: Akt1
50
IB: ERK-p42/p44
40
IB: ERK
40
IB: Vinculin
100
Fig. S1g
IB: Akt-pT308
KD
50
IB: Akt1
50
IB: ERK-p42/p44
40
IB: ERK
40
IB: Vinculin
100
Fig. S1h
IB: Akt-pT308
KD
50
IB: Akt1
50
IB: ERK-p42/p44
40
IB: ERK
40
IB: Vinculin
100
Fig. S1i
IB: Akt-pT308
KD
50
IB: Akt1
50
IB: ERK-p42/p44
40
IB: ERK
40
IB: Vinculin
100
Fig. S1j
IB: Akt-pT308
KD
50
50
IB: Akt1
IB: ERK-p42/p44
40
IB: ERK
40
IB: Vinculin
100
Fig. S1k
IB: Akt-pT308
KD
50
IB: Akt1
50
IB: ERK-p42/p44
40
IB: ERK
40
IB: Vinculin
100

Fig. S2d

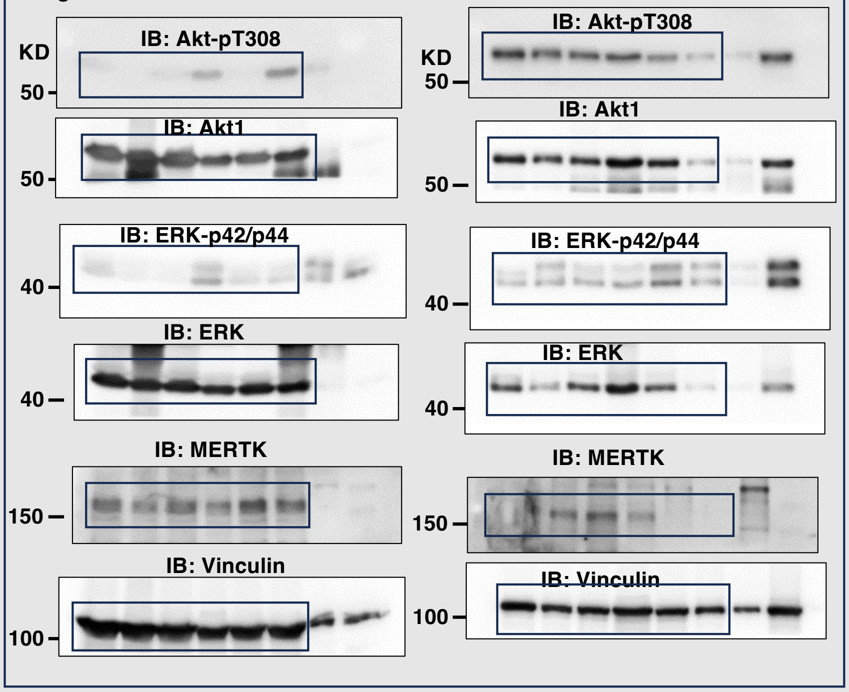

KD
IB: Akt-pT308
50
IB: Akt1
50
IB: ERK-p42/p44
40
IB: ERK
40
IB: MERTK
150
IB: Vinculin
100
KD
IB: Akt-pT308
50
IB: Akt1
50
IB: ERK-p42/p44
40
IB: ERK
40
IB: MERTK
150
IB: Vinculin
100

Fig. S3a

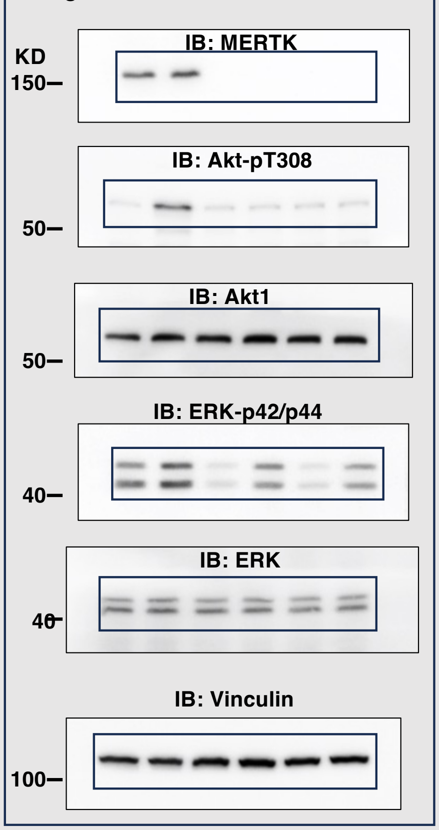

Fig. S3b

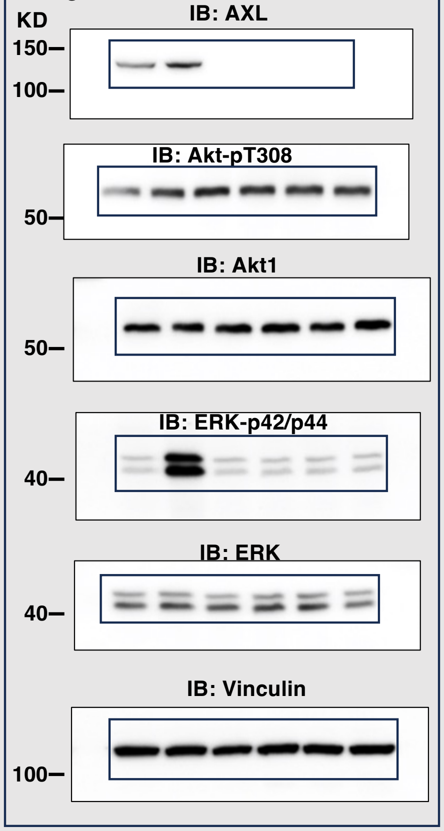

Fig. S3c

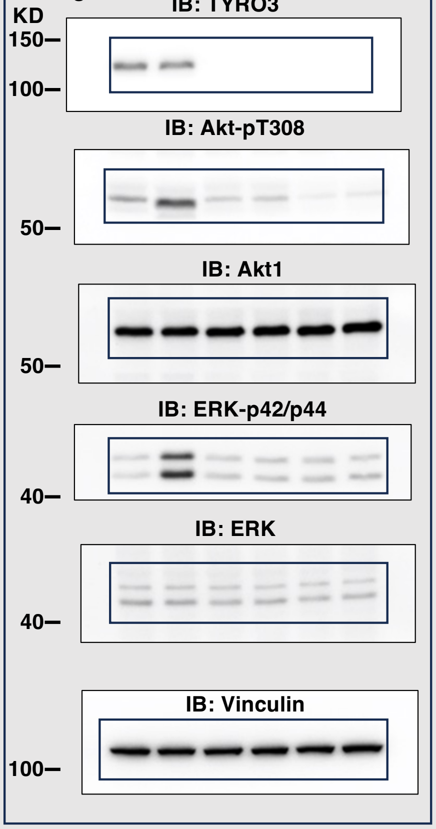

Fig. S3d

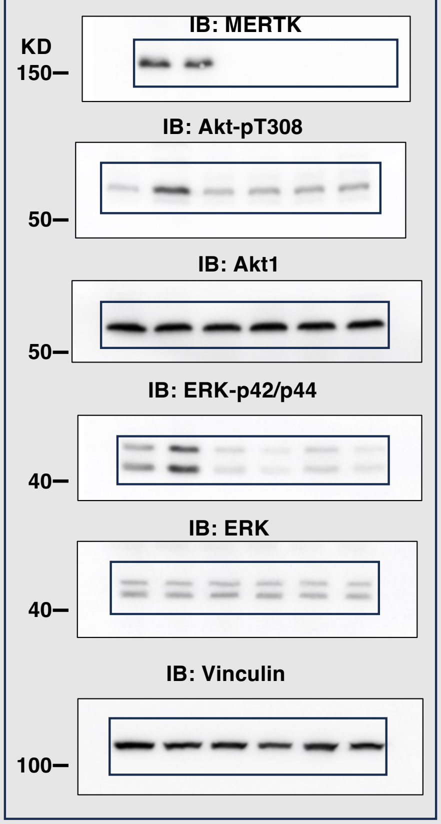

Fig. S3e

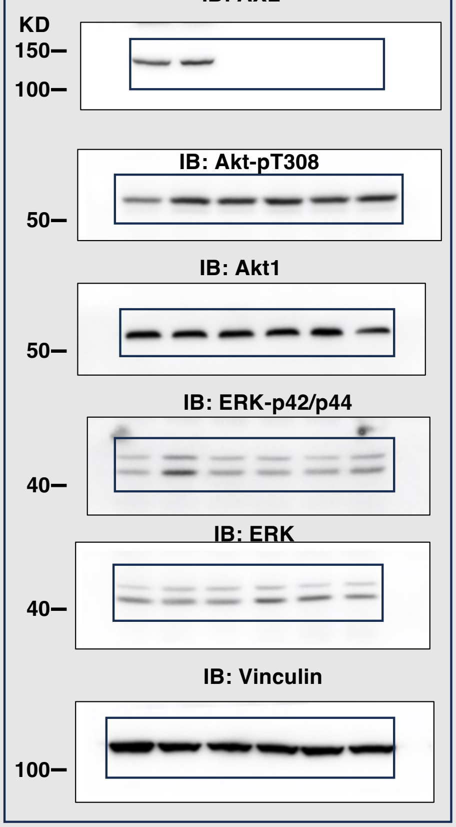

Fig. S3f

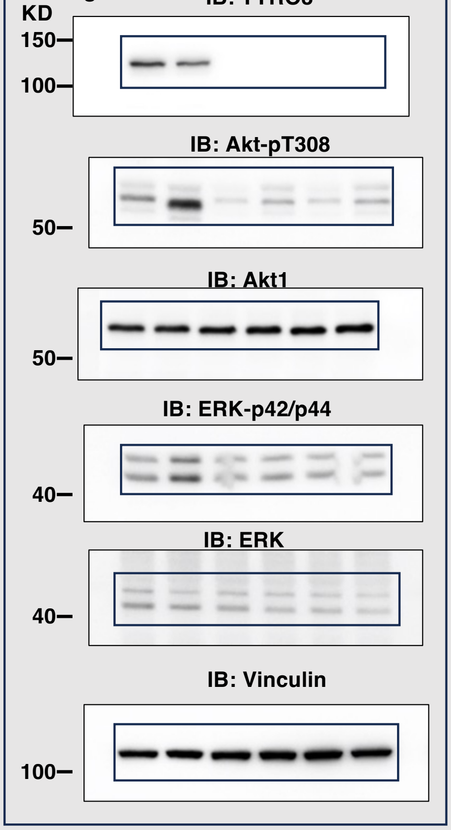

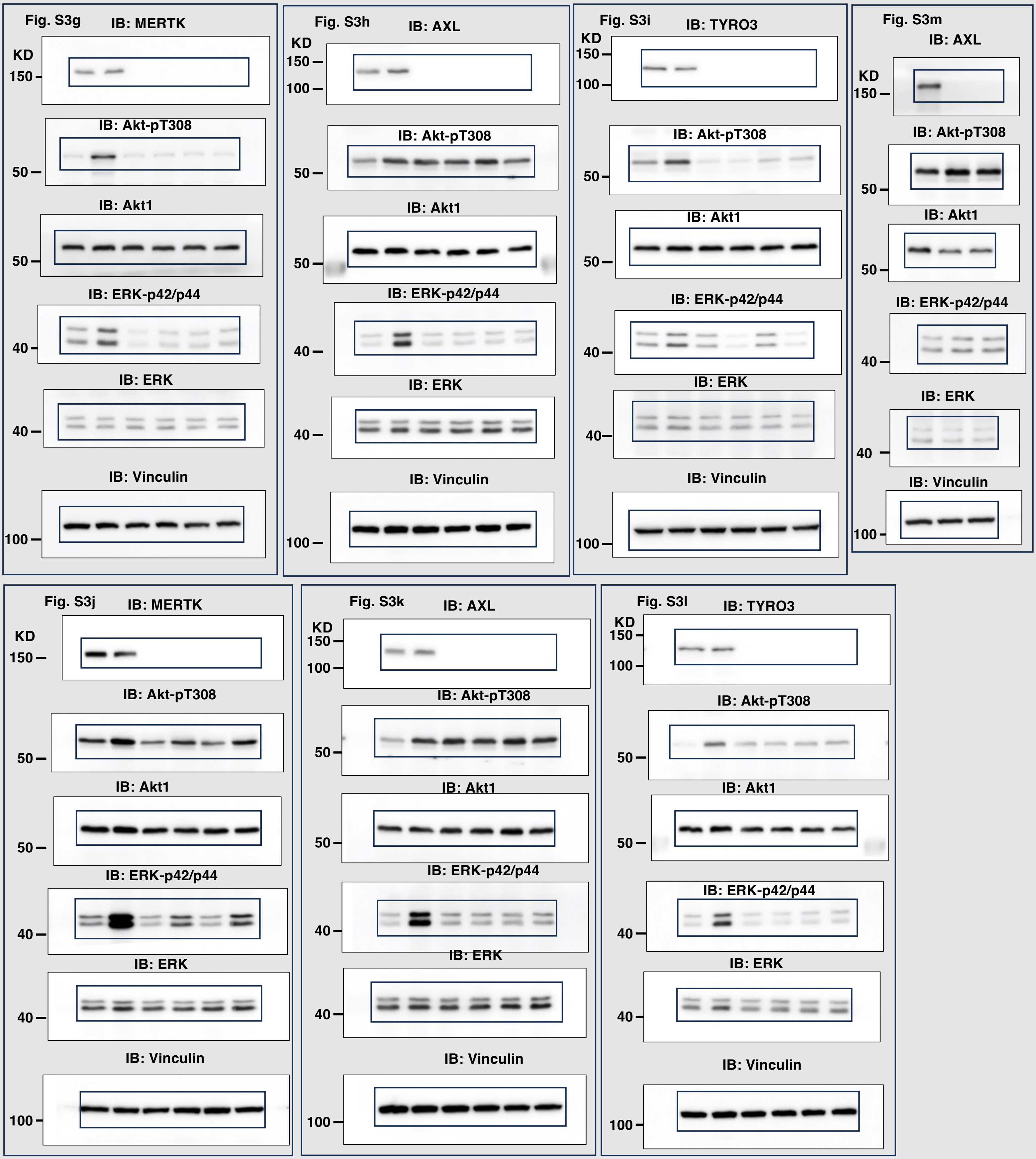
Fig. S3g
IB: MERTK
KD
150
IB: Akt-pT308
50
IB: Akt1
50
IB: ERK-p42/p44
40
IB: ERK
40
IB: Vinculin
100
Fig. S3h
IB: AXL
KD
150
100
IB: Akt-pT308
50
IB: Akt1
50
IB: ERK-p42/p44
40
IB: ERK
40
IB: Vinculin
100
Fig. S3i
IB: TYRO3
KD
150
100
IB: Akt-pT308
50
IB: Akt1
50
IB: ERK-p42/p44
40
IB: ERK
40
IB: Vinculin
100
Fig. S3m
IB: AXL
KD
150
IB: Akt-pT308
50
IB: Akt1
50
IB: ERK-p42/p44
40
IB: ERK
40
IB: Vinculin
100
Fig. S3j
IB: MERTK
KD
150
IB: Akt-pT308
50
IB: Akt1
50
IB: ERK-p42/p44
40
IB: ERK
40
IB: Vinculin
100
Fig. S3k
IB: AXL
KD
150
100
IB: Akt-pT308
50
IB: Akt1
50
IB: ERK-p42/p44
40
IB: ERK
40
IB: Vinculin
100
Fig. S3l
IB: TYRO3
KD
150
100
IB: Akt-pT308
50
IB: Akt1
50
IB: ERK-p42/p44
40
IB: ERK
40
IB: Vinculin
100

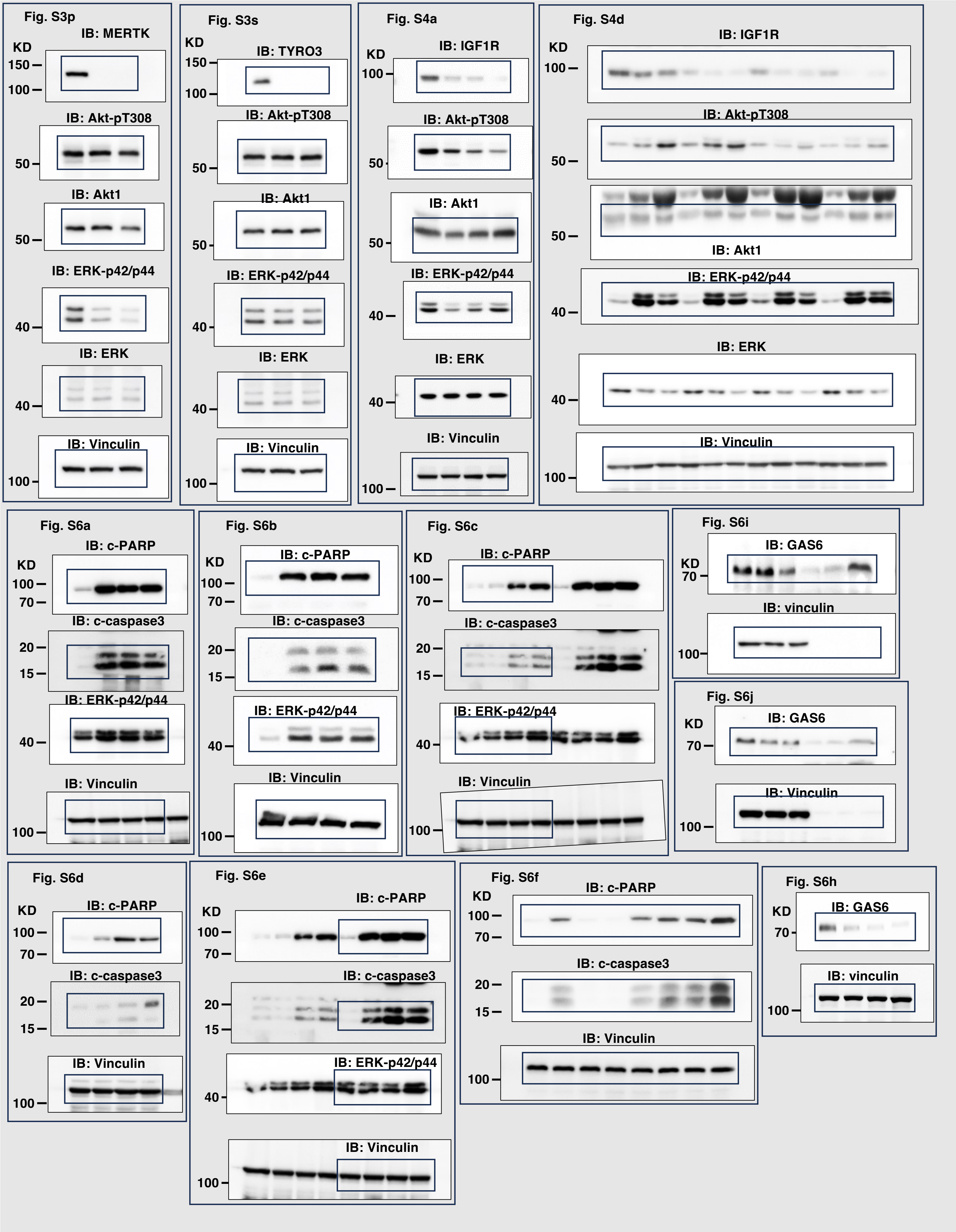
Fig. S3p
IB: MERTK
KD
150
100
IB: Akt-pT308
50
IB: Akt1
50
IB: ERK-p42/p44
40
IB: ERK
40
IB: Vinculin
100
Fig. S3s
KD
IB: TYRO3
150
100
IB: Akt-pT308
50
IB: Akt1
50
IB: ERK-p42/p44
40
IB: ERK
40
IB: Vinculin
100
Fig. S4a
KD
IB: IGF1R
100
IB: Akt-pT308
50
IB: Akt1
50
IB: ERK-p42/p44
40
IB: ERK
40
IB: Vinculin
100
Fig. S4d
KD
IB: IGF1R
100
IB: Akt-pT308
50
50
IB: Akt1
IB: ERK-p42/p44
40
IB: ERK
40
IB: Vinculin
100
Fig. S6a
KD
IB: c-PARP
100
70
IB: c-caspase3
20
15
IB: ERK-p42/p44
40
IB: Vinculin
100
Fig. S6b
KD
IB: c-PARP
100
70
IB: c-caspase3
20
15
IB: ERK-p42/p44
40
IB: Vinculin
100
Fig. S6c
KD
IB: c-PARP
100
70
IB: c-caspase3
20
15
IB: ERK-p42/p44
40
IB: Vinculin
100
Fig. S6i
IB: GAS6
KD
70
IB: vinculin
100
Fig. S6j
KD
IB: GAS6
70
IB: Vinculin
100
Fig. S6d
KD
IB: c-PARP
100
70
IB: c-caspase3
20
15
IB: Vinculin
100
Fig. S6e
KD
IB: c-PARP
100
70
IB: c-caspase3
20
15
IB: ERK-p42/p44
40
IB: Vinculin
100
Fig. S6f
KD
IB: c-PARP
100
70
IB: c-caspase3
20
15
IB: Vinculin
100
Fig. S6h
KD
IB: GAS6
70
IB: vinculin
100

Fig. S7a

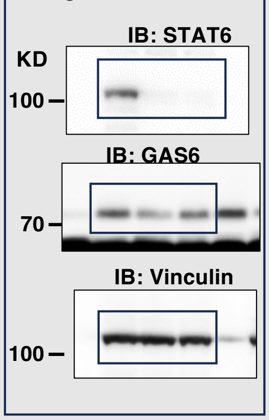

Fig. S7b

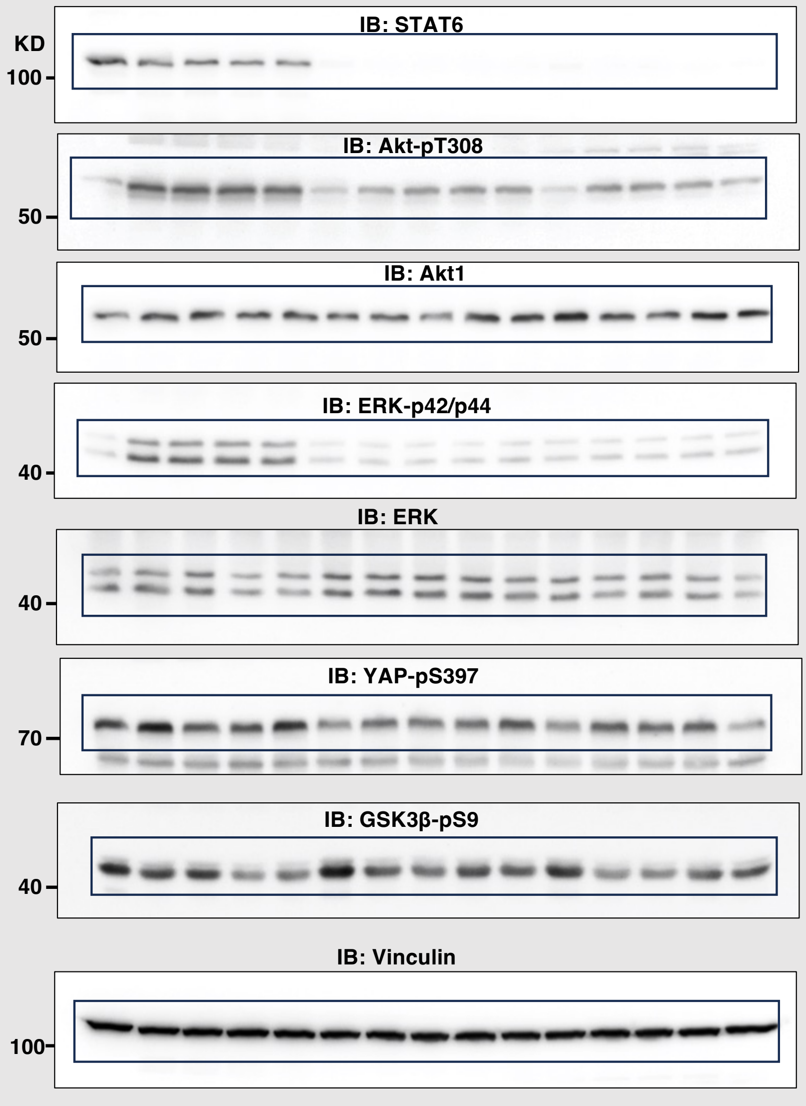

Fig. S7c

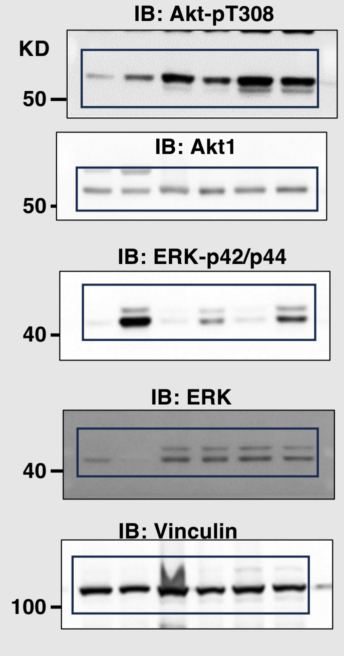

Fig. S8a

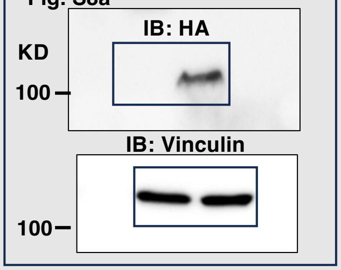

Fig. S8b

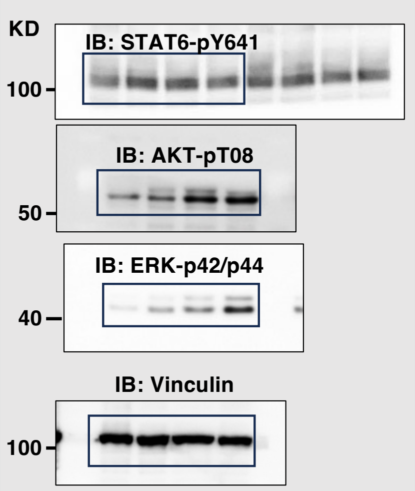

Fig. S8c

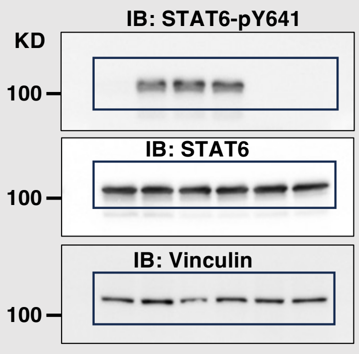

Fig. S8d

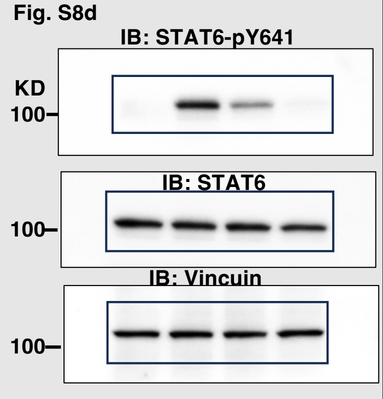

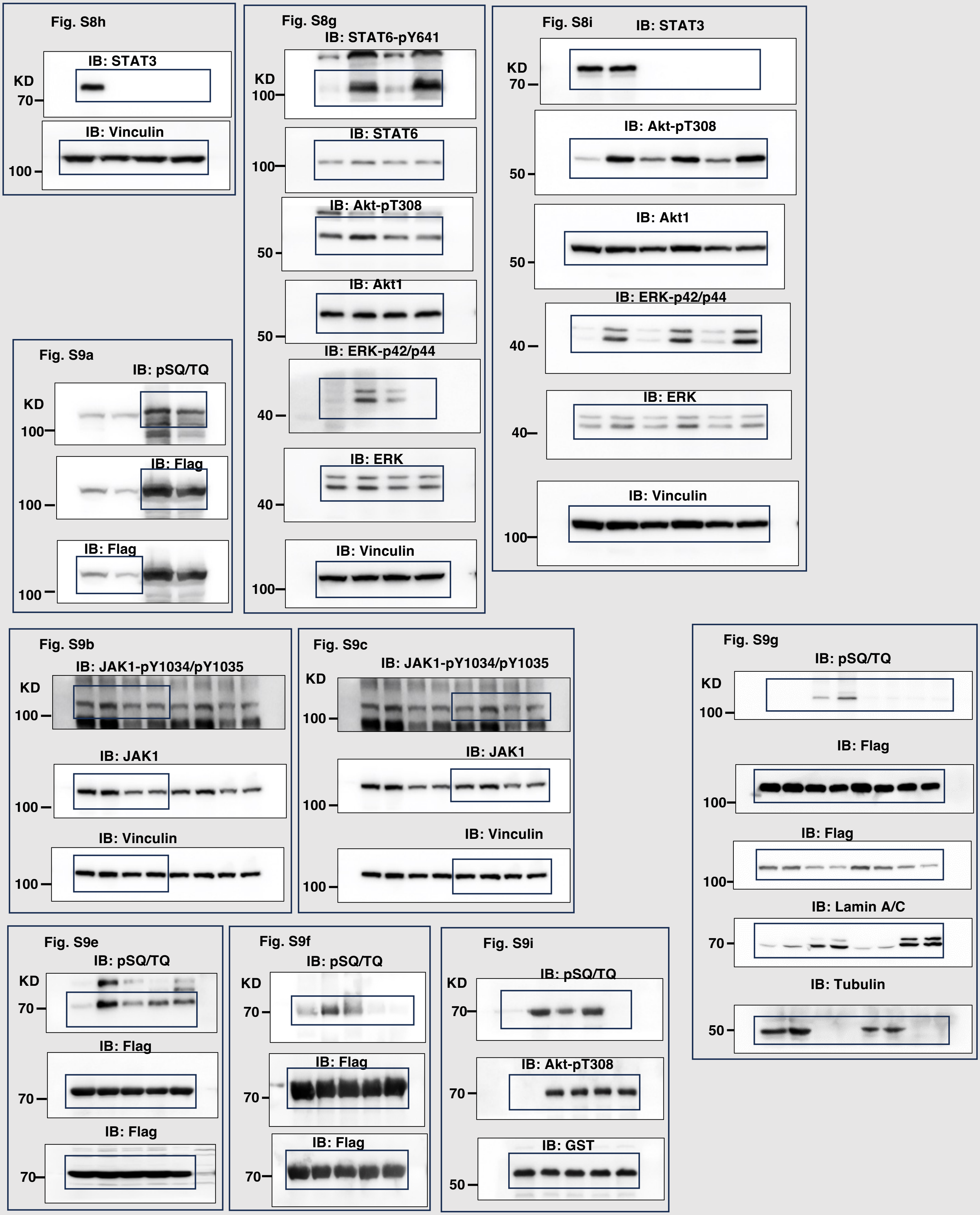
Fig. S8h
IB: STAT3
KD
70
IB: Vinculin
100
Fig. S8g
IB: STAT6-pY641
KD
100
IB: STAT6
100
IB: Akt-pT308
50
IB: Akt1
50
IB: ERK-p42/p44
40
IB: ERK
40
IB: Vinculin
100
Fig. S8i
IB: STAT3
KD
70
IB: Akt-pT308
50
IB: Akt1
50
IB: ERK-p42/p44
40
IB: ERK
40
IB: Vinculin
100
Fig. S9a
IB: pSQ/TQ
KD
100
IB: Flag
100
IB: Flag
100
Fig. S9b
IB: JAK1-pY1034/pY1035
KD
100
IB: JAK1
100
IB: Vinculin
100
Fig. S9c
IB: JAK1-pY1034/pY1035
KD
100
IB: JAK1
100
IB: Vinculin
100
Fig. S9g
IB: pSQ/TQ
KD
100
IB: Flag
100
IB: Flag
100
IB: Lamin A/C
70
IB: Tubulin
50
Fig. S9e
IB: pSQ/TQ
KD
70
IB: Flag
70
IB: Flag
70
Fig. S9f
IB: pSQ/TQ
KD
70
IB: Flag
70
IB: Flag
70
Fig. S9i
IB: pSQ/TQ
KD
70
IB: Akt-pT308
70
IB: GST
50
