# Supplementary material for: Co-targeting JAK1/STAT6/GAS6/TAM signaling improves chemotherapy efficacy in Ewing sarcoma
Source: Nat Commun. 2024 Jun 21;15:5292. doi: 10.1038/s41467-024-49667-2 (PMC11192891; doi:10.1038/s41467-024-49667-2)
Supplement: Supplementary file 2 — Reporting Summary [file 41467_2024_49667_MOESM2_ESM.pdf]

nature portfolio

Corresponding author(s): Pengda Liu

Last updated by author(s): May 10, 2024

Reporting Summary

Nature Portfolio wishes to improve the reproducibility of the work that we publish. This form provides structure for consistency and transparency in reporting. For further information on Nature Portfolio policies, see our Editorial Policies and the Editorial Policy Checklist.

Statistics

For all statistical analyses, confirm that the following items are present in the figure legend, table legend, main text, or Methods section.

n/a	Confirmed	
[ ]	[x]	The exact sample size (*n*) for each experimental group/condition, given as a discrete number and unit of measurement
[ ]	[x]	A statement on whether measurements were taken from distinct samples or whether the same sample was measured repeatedly
[ ]	[x]	The statistical test(s) used AND whether they are one- or two-sided<br>*Only common tests should be described solely by name; describe more complex techniques in the Methods section.*
[x]	[ ]	A description of all covariates tested
[x]	[ ]	A description of any assumptions or corrections, such as tests of normality and adjustment for multiple comparisons
[ ]	[x]	A full description of the statistical parameters including central tendency (e.g. means) or other basic estimates (e.g. regression coefficient) AND variation (e.g. standard deviation) or associated estimates of uncertainty (e.g. confidence intervals)
[ ]	[x]	For null hypothesis testing, the test statistic (e.g. *F*, *t*, *r*) with confidence intervals, effect sizes, degrees of freedom and *P* value noted<br>*Give P values as exact values whenever suitable.*
[x]	[ ]	For Bayesian analysis, information on the choice of priors and Markov chain Monte Carlo settings
[x]	[ ]	For hierarchical and complex designs, identification of the appropriate level for tests and full reporting of outcomes
[x]	[ ]	Estimates of effect sizes (e.g. Cohen's *d*, Pearson's *r*), indicating how they were calculated

Our web collection on statistics for biologists contains articles on many of the points above.

Software and code

Policy information about availability of computer code

Data collection	N/A
Data analysis	N/A

For manuscripts utilizing custom algorithms or software that are central to the research but not yet described in published literature, software must be made available to editors and reviewers. We strongly encourage code deposition in a community repository (e.g. GitHub). See the Nature Portfolio guidelines for submitting code & software for further information.

Data

Policy information about availability of data

All manuscripts must include a data availability statement. This statement should provide the following information, where applicable:

- Accession codes, unique identifiers, or web links for publicly available datasets
- A description of any restrictions on data availability
- For clinical datasets or third party data, please ensure that the statement adheres to our policy

Original scans of uncropped and unprocessed western blot images are available and submitted to journal as a source data file. All quantification data for plots are also included and submitted as a source data file. All data and materials used in the analysis are available upon reasonable request.

Research involving human participants, their data, or biological material

Policy information about studies with human participants or human data. See also policy information about sex, gender (identity/presentation), and sexual orientation and race, ethnicity and racism.

Reporting on sex and gender	N/A
Reporting on race, ethnicity, or other socially relevant groupings	N/A
Population characteristics	N/A
Recruitment	N/A
Ethics oversight	N/A

Note that full information on the approval of the study protocol must also be provided in the manuscript.

Field-specific reporting

Please select the one below that is the best fit for your research. If you are not sure, read the appropriate sections before making your selection.

☒ Life sciences ☐ Behavioural & social sciences ☐ Ecological, evolutionary & environmental sciences

For a reference copy of the document with all sections, see nature.com/documents/nr-reporting-summary-flat.pdf

Life sciences study design

All studies must disclose on these points even when the disclosure is negative.

Sample size	Sample sizes were not predetermined and were indicated in the corresponding figure legends. For colony formation and cell viability assays, at least 3 biological repeats were performed to ensure reproducibility. For animal studies, 5 mice per group were chosen based on previous common practice in the field, and animal welfare guidelines and availability of animals, while minimizing the use of animals in accordance with guidelines from the University of North Carolina at Chapel Hill Institutional Animal Care and Use Committee.
Data exclusions	No data exclusion in this study.
Replication	Data presented are representatives from at least biological replicates to ensure reproducibility. Experimental findings were also repeated by multiple methods including repetitions of the exact experimental conditions, validation of findings using additional cell lines or approaches, using both in vitro and in vivo models. Number of replications indicate on figure legends and experiments were successfully replicated.
Randomization	Cell culture experiments were pooled before splitting into individual identical wells before being randomly assigned for each experimental conditions. Mice were randomly grouped into indicated groups for these treatment experiments.
Blinding	Experiments were performed blinded when possible. Blinding was not possible for many experiments as especially for cell culture experiments given experimental conditions had to be monitored throughout the experiment. To mitigate this, data were plotted and analyzed at the end of the experiment and measurements and analysis were confirmed independently.

Reporting for specific materials, systems and methods

We require information from authors about some types of materials, experimental systems and methods used in many studies. Here, indicate whether each material, system or method listed is relevant to your study. If you are not sure if a list item applies to your research, read the appropriate section before selecting a response.

Materials & experimental systems

n/a	Involved in the study	
☐	☒	Antibodies
☐	☒	Eukaryotic cell lines
☒	☐	Palaeontology and archaeology
☐	☒	Animals and other organisms
☒	☐	Clinical data
☒	☐	Dual use research of concern
☒	☐	Plants

Methods

n/a	Involved in the study	
☒	☐	ChIP-seq
☒	☐	Flow cytometry
☒	☐	MRI-based neuroimaging

Antibodies

Antibodies used	Anti-pT308-AKT antibody (2965), anti-AKT1 antibody (2938), anti-GST antibody (2625), anti-p42/44-ERK (4370), Anti-HA antibody (3724), GAS6 (67202), c-PARP (5625), c-caspase3 (672029661), STAT6 (9362), STAT6-pY641 (9364), TEAD1 (12292), pSQ/TQ (2851), anti-rabbit IgG, HRP-linked antibody (7074) and anti-mouse IgG, HRP-linked antibody (7076), were obtained from Cell Signaling Technology. anti-ERK (sc-135900), anti-AXL antibody (sc-166268), anti-TYRO3 antibody (sc-166359), Anti-GST antibody (sc-138), Lamin A/C (sc-7293), anti-vinculin antibody (sc-25336) were obtained from Santa Cruz Biotechnology. Polyclonal anti-Flag antibody (F-7425), monoclonal anti-Flag antibody (F-3165, clone M2) were obtained from Sigma. anti-Flag agarose beads (A-2220), anti-HA agarose beads (A-2095) were obtained from Sigma. Anti-MERTK antibody was generated by the Shelton Earp lab at UNC-CH.
Validation	All commercially available antibodies were verified according to the manufacturer's specifications. Some antibodies such as MERTK, AXL, TYRO3, STAT6, JAK1 and GAS6 were validated using knockdown cell lines.

Eukaryotic cell lines

Policy information about cell lines and Sex and Gender in Research

Cell line source(s)	Ewing sarcoma cells used in this study were obtained either from Dr. Ian Davis lab at UNC or COG (childrens' oncology group). Cells have been continuously monitored for mycoplasma contamination. The Ewing sarcoma PDX tumor NCH-EWS-1 was obtained from Dr. Peter J. Houghton at UT Health San Antonio.
Authentication	A673, MHH-ES-1 and SK-N-MC cells were authenticated by STR recently. All other cell lines were verified by the manufacturer's websites and regularly checked by morphology.
Mycoplasma contamination	Cell lines were not tested for mycoplasma contamination but constantly monitored during cell culture.
Commonly misidentified lines (See ICLAC register)	N/A

Animals and other research organisms

Policy information about studies involving animals; ARRIVE guidelines recommended for reporting animal research, and Sex and Gender in Research

Laboratory animals	Nude mice (Jackson Lab 002019) are used in all animal experiments in this manuscript.
Wild animals	N/A
Reporting on sex	Sex has been considered and female nude mice have been used in different experiments.
Field-collected samples	N/A
Ethics oversight	All mouse work has been reviewed and approved by UNC Institutional Animal Care and Use Committee under IACUC#22-056.

Note that full information on the approval of the study protocol must also be provided in the manuscript.

Plants

Seed stocks	N/A
Novel plant genotypes	N/A
Authentication	N/A
