# Supplementary material for: Co-targeting JAK1/STAT6/GAS6/TAM signaling improves chemotherapy efficacy in Ewing sarcoma
Source: Nat Commun. 2024 Jun 21;15:5292. doi: 10.1038/s41467-024-49667-2 (PMC11192891; doi:10.1038/s41467-024-49667-2)
Supplement: Supplementary file 4 — Source Data [file 41467_2024_49667_MOESM4_ESM.zip › Source Data uncropped original images for immuno blots.pptx]

## Slide 1
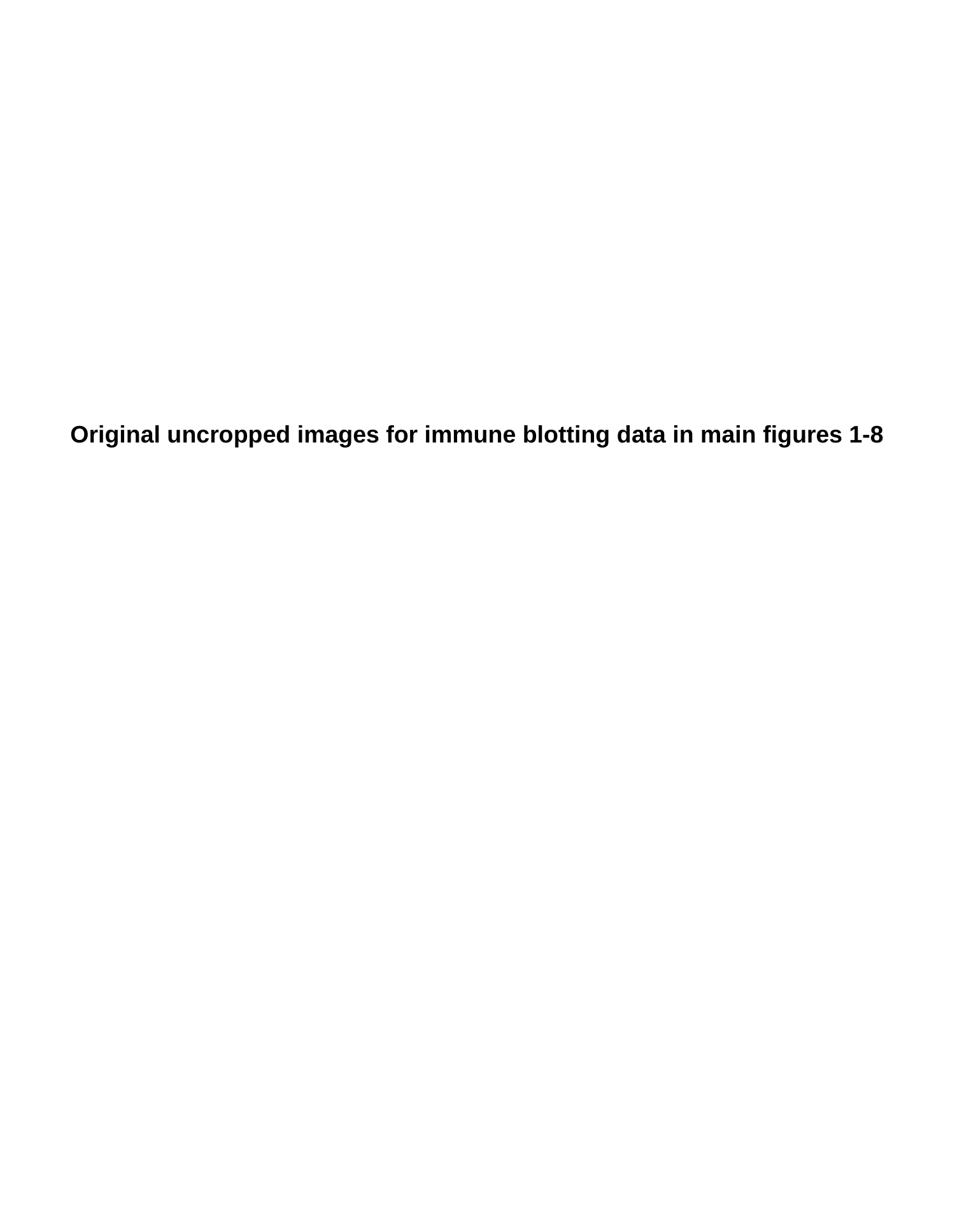

Original uncropped images for immune blotting data in main figures 1-8

## Slide 2
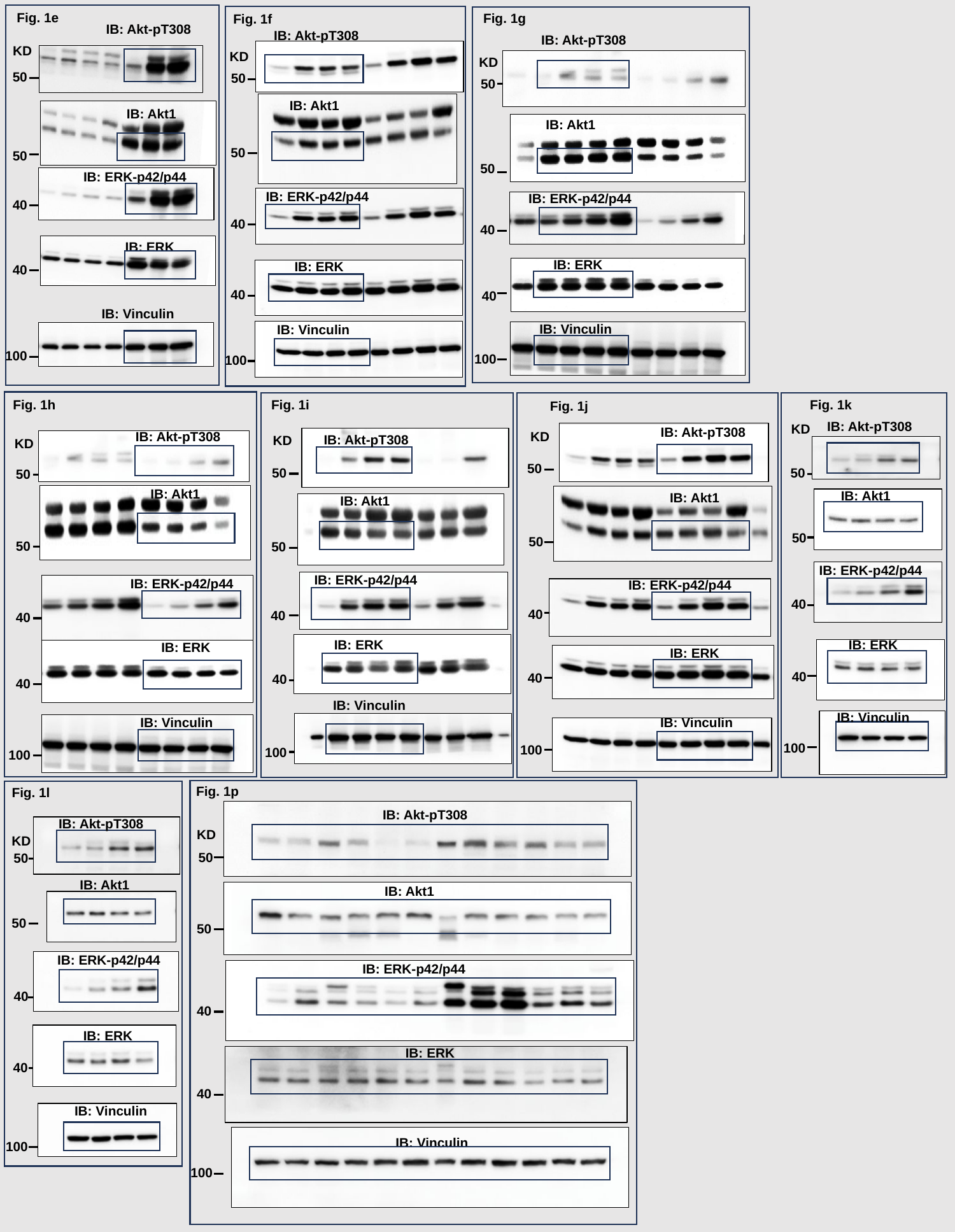

Fig. 1e
IB: Akt-pT308
KD
50
IB: Akt1
50
IB: ERK-p42/p44
40
IB: ERK
40
IB: Vinculin
100
Fig. 1g
IB: Akt-pT308
KD
50
IB: Akt1
50
IB: ERK-p42/p44
40
IB: ERK
40
IB: Vinculin
100
Fig. 1f
IB: Akt-pT308
IB: Akt1
IB: ERK-p42/p44
IB: ERK
IB: Vinculin
KD
50
50
40
40
100
Fig. 1h
IB: Akt-pT308
IB: Akt1
IB: ERK-p42/p44
IB: ERK
IB: Vinculin
Fig. 1i
Fig. 1k
IB: Akt-pT308
IB: Akt1
IB: ERK-p42/p44
IB: ERK
IB: Vinculin
Fig. 1j
IB: Akt-pT308
IB: Akt1
IB: ERK-p42/p44
IB: ERK
IB: Vinculin
KD
KD
IB: Akt-pT308
KD
KD
50
50
50
50
IB: Akt1
50
50
50
50
IB: ERK-p42/p44
40
40
40
40
IB: ERK
40
40
40
40
IB: Vinculin
100
100
100
100
Fig. 1p
IB: Akt-pT308
IB: Akt1
IB: ERK-p42/p44
IB: ERK
IB: Vinculin
Fig. 1l
IB: Akt-pT308
IB: Akt1
IB: ERK-p42/p44
IB: ERK
IB: Vinculin
KD
KD
50
50
50
50
40
40
40
40
100
100

## Slide 3
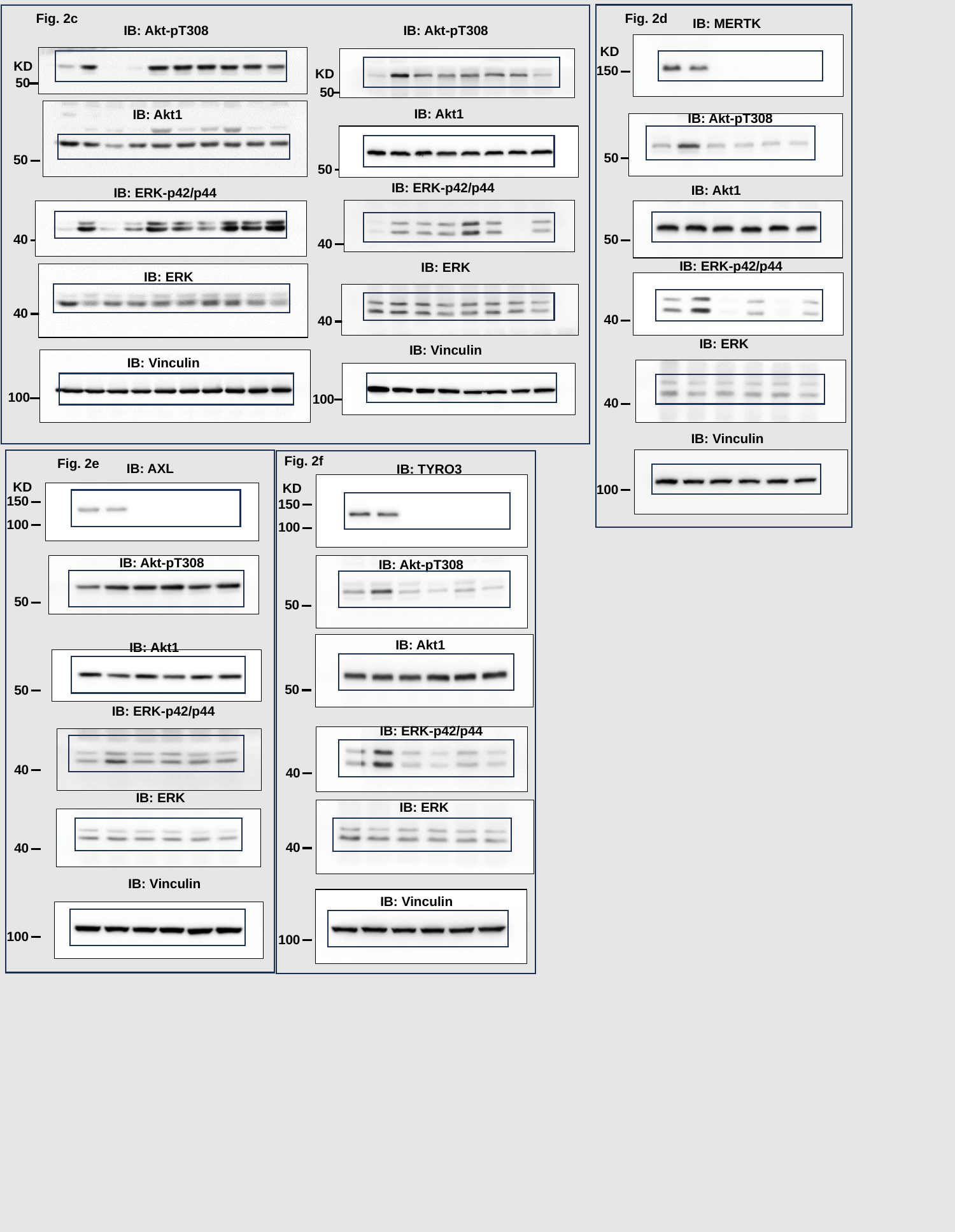

Fig. 2d
IB: MERTK
IB: Akt-pT308
IB: Akt1
IB: ERK-p42/p44
IB: ERK
IB: Vinculin
Fig. 2c
IB: Akt-pT308
IB: Akt-pT308
KD
KD
150
KD
50
50
IB: Akt1
IB: Akt1
50
50
50
IB: ERK-p42/p44
IB: ERK-p42/p44
40
50
40
IB: ERK
IB: ERK
40
40
40
IB: Vinculin
IB: Vinculin
100
100
40
Fig. 2f
IB: TYRO3
IB: Akt-pT308
IB: Akt1
IB: ERK-p42/p44
IB: ERK
IB: Vinculin
Fig. 2e
IB: AXL
IB: Akt-pT308
IB: Akt1
IB: ERK-p42/p44
IB: ERK
IB: Vinculin
KD
KD
100
150
150
100
100
50
50
50
50
40
40
40
40
100
100

## Slide 4
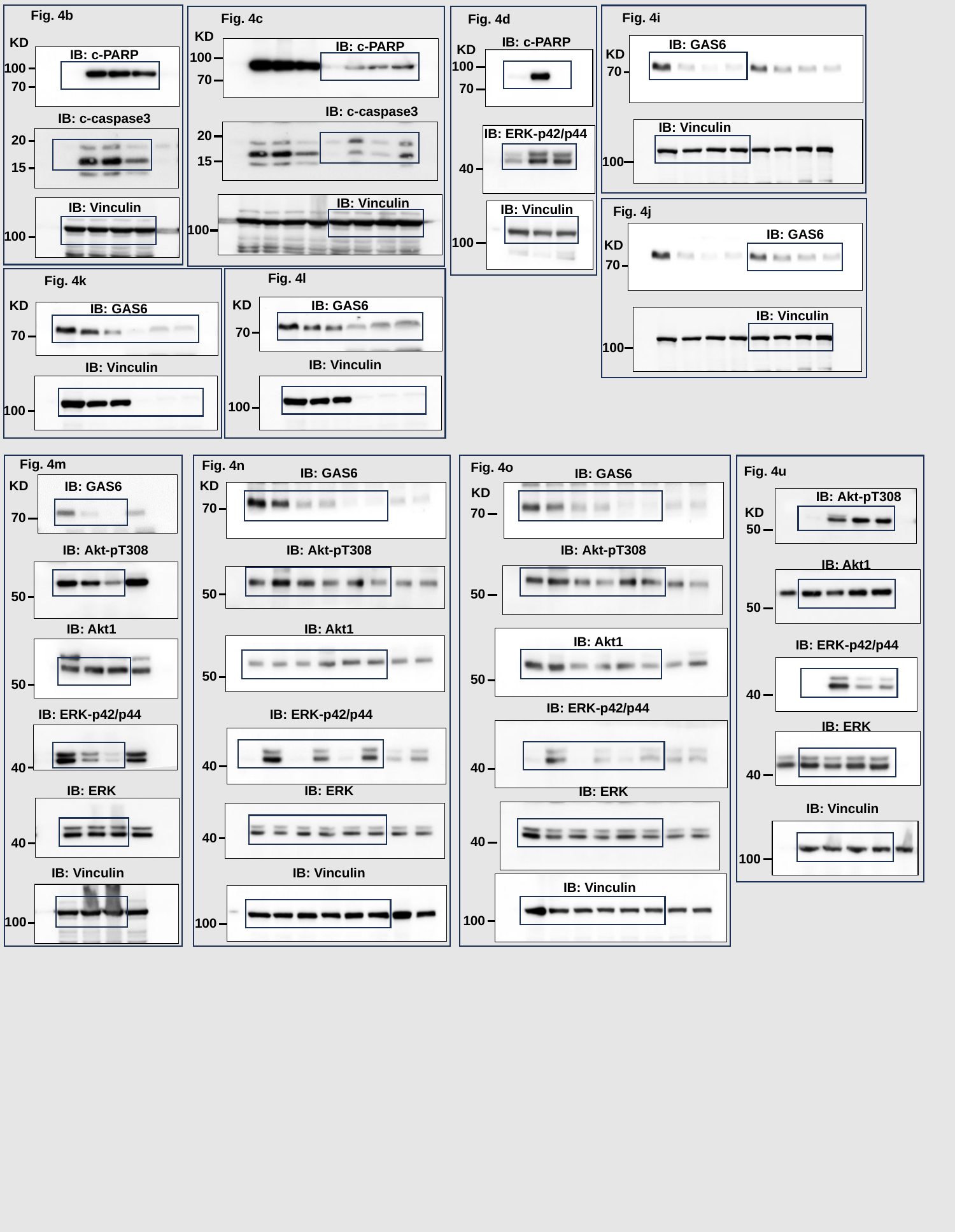

Fig. 4b
IB: c-PARP
IB: c-caspase3
IB: Vinculin
Fig. 4i
IB: GAS6
IB: Vinculin
Fig. 4c
IB: c-PARP
IB: c-caspase3
IB: Vinculin
Fig. 4d
KD
IB: c-PARP
KD
KD
KD
100
100
100
70
70
70
70
IB: ERK-p42/p44
20
20
15
100
15
40
IB: Vinculin
Fig. 4j
IB: GAS6
IB: Vinculin
100
100
100
KD
70
Fig. 4l
IB: GAS6
IB: Vinculin
Fig. 4k
IB: GAS6
IB: Vinculin
KD
KD
70
70
100
100
100
Fig. 4m
IB: GAS6
IB: Akt-pT308
IB: Akt1
IB: ERK-p42/p44
IB: ERK
IB: Vinculin
Fig. 4n
IB: GAS6
IB: Akt-pT308
IB: Akt1
IB: ERK-p42/p44
IB: ERK
IB: Vinculin
Fig. 4o
IB: GAS6
IB: Akt-pT308
IB: Akt1
IB: ERK-p42/p44
IB: ERK
IB: Vinculin
Fig. 4u
IB: Akt-pT308
IB: Akt1
IB: ERK-p42/p44
IB: ERK
IB: Vinculin
KD
KD
KD
70
KD
70
70
50
50
50
50
50
50
50
50
40
40
40
40
40
40
40
40
100
100
100
100

## Slide 5
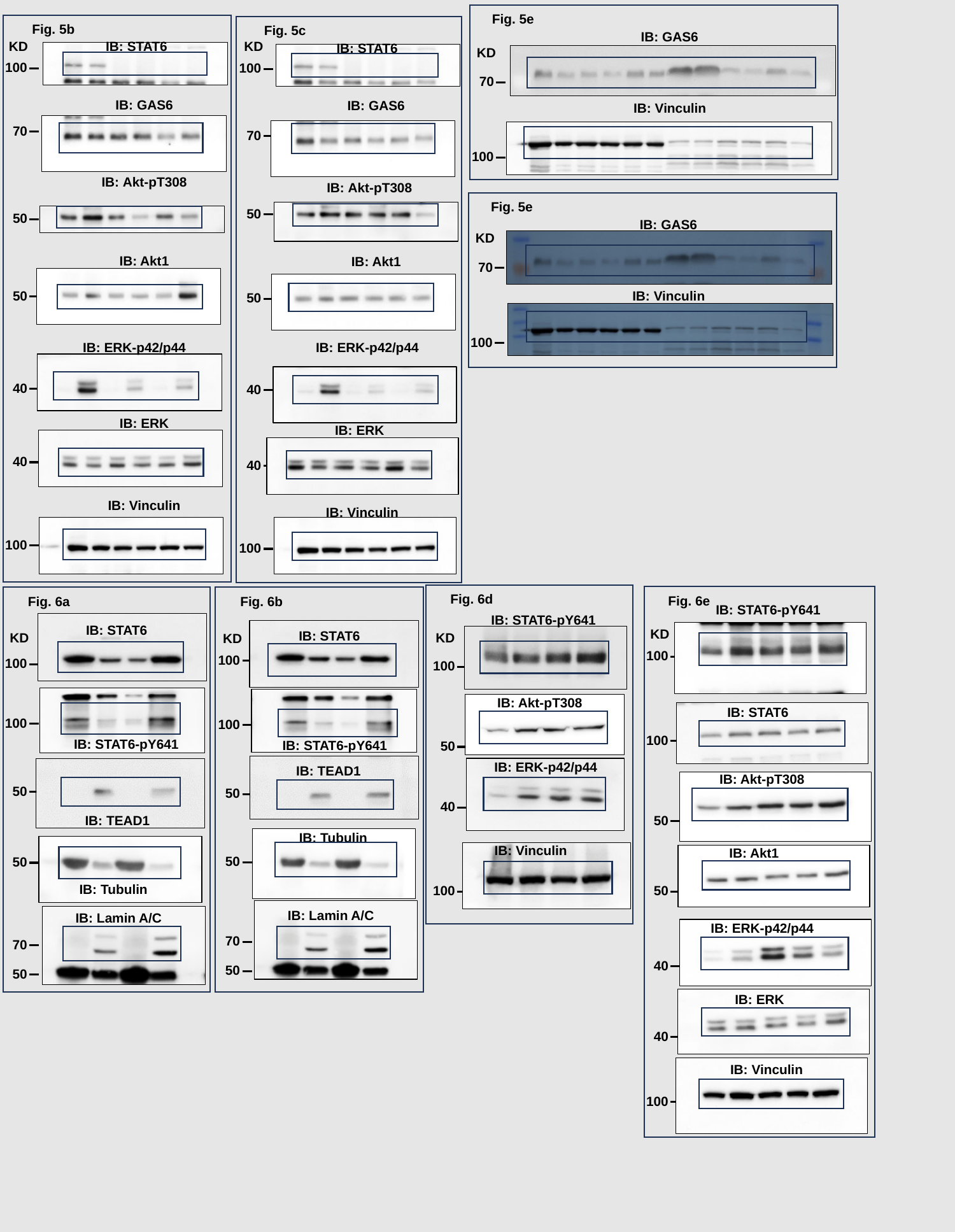

Fig. 5e
IB: GAS6
IB: Vinculin
Fig. 5b
IB: STAT6
IB: GAS6
IB: Akt-pT308
IB: Akt1
IB: ERK-p42/p44
IB: ERK
IB: Vinculin
Fig. 5c
IB: STAT6
IB: GAS6
IB: Akt-pT308
IB: Akt1
IB: ERK-p42/p44
IB: ERK
IB: Vinculin
KD
KD
KD
100
100
70
70
70
100
Fig. 5e
IB: GAS6
IB: Vinculin
50
50
KD
70
50
50
100
40
40
40
40
100
100
Fig. 6d
IB: STAT6-pY641
IB: Akt-pT308
IB: ERK-p42/p44
IB: Vinculin
Fig. 6e
IB: STAT6-pY641
IB: STAT6
IB: Akt-pT308
IB: Akt1
IB: ERK-p42/p44
IB: ERK
IB: Vinculin
Fig. 6b
IB: STAT6
IB: STAT6-pY641
IB: TEAD1
IB: Tubulin
IB: Lamin A/C
Fig. 6a
IB: STAT6
IB: STAT6-pY641
IB: TEAD1
IB: Tubulin
IB: Lamin A/C
KD
KD
KD
KD
100
100
100
100
100
100
100
50
50
50
40
50
50
50
100
50
70
70
40
50
50
40
100

## Slide 6
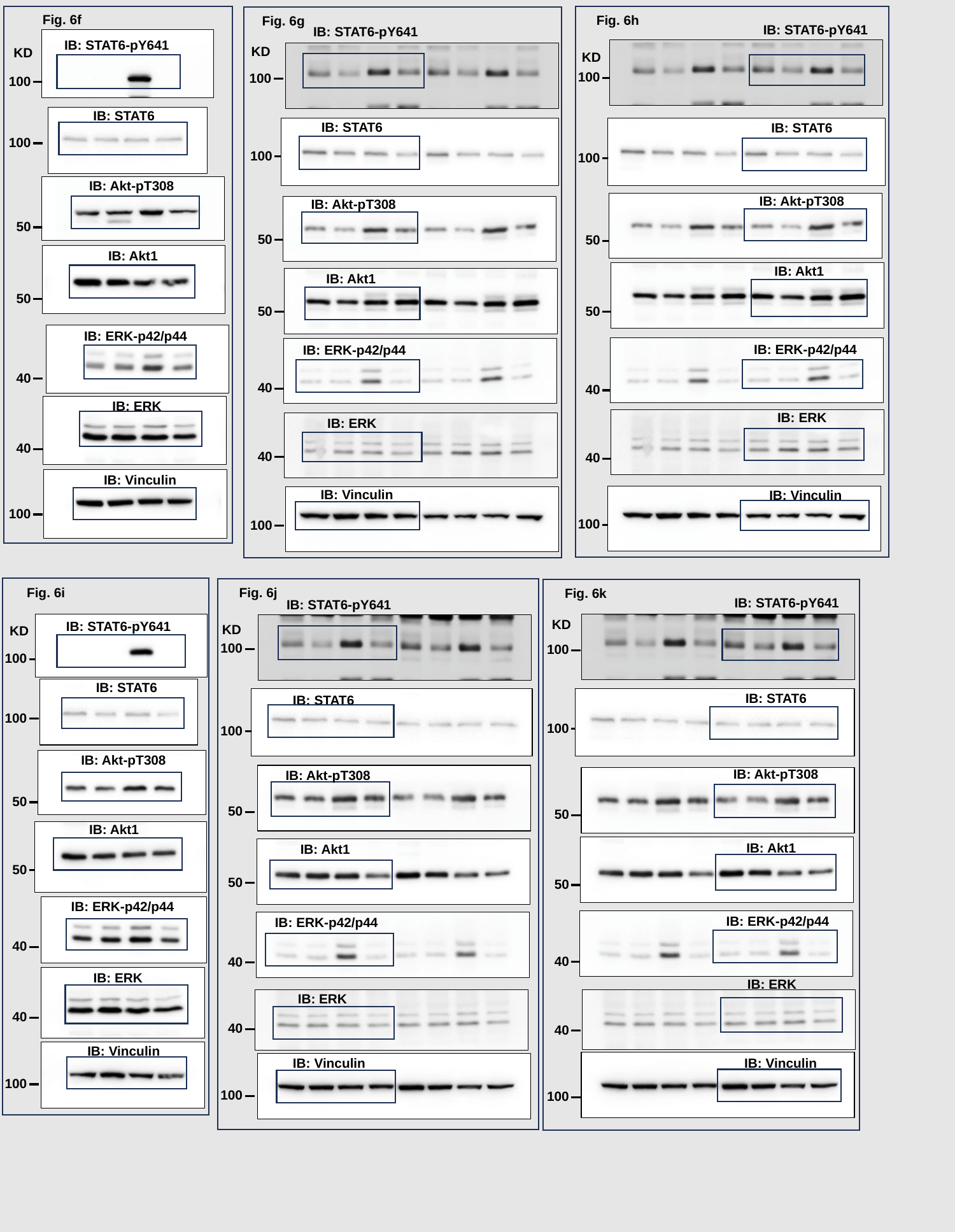

Fig. 6h
IB: STAT6-pY641
IB: STAT6
IB: Akt-pT308
IB: Akt1
IB: ERK-p42/p44
IB: ERK
IB: Vinculin
Fig. 6f
Fig. 6g
IB: STAT6-pY641
IB: STAT6
IB: Akt-pT308
IB: Akt1
IB: ERK-p42/p44
IB: ERK
IB: Vinculin
IB: STAT6-pY641
KD
KD
KD
100
100
100
IB: STAT6
100
100
100
IB: Akt-pT308
50
50
50
IB: Akt1
50
50
50
IB: ERK-p42/p44
40
40
40
IB: ERK
40
40
40
IB: Vinculin
100
100
100
Fig. 6j
IB: STAT6-pY641
IB: STAT6
IB: Akt-pT308
IB: Akt1
IB: ERK-p42/p44
IB: ERK
IB: Vinculin
Fig. 6k
IB: STAT6-pY641
IB: STAT6
IB: Akt-pT308
IB: Akt1
IB: ERK-p42/p44
IB: ERK
IB: Vinculin
Fig. 6i
KD
IB: STAT6-pY641
KD
KD
100
100
100
IB: STAT6
100
100
100
IB: Akt-pT308
50
50
50
IB: Akt1
50
50
50
IB: ERK-p42/p44
40
40
40
IB: ERK
40
40
40
IB: Vinculin
100
100
100

## Slide 7
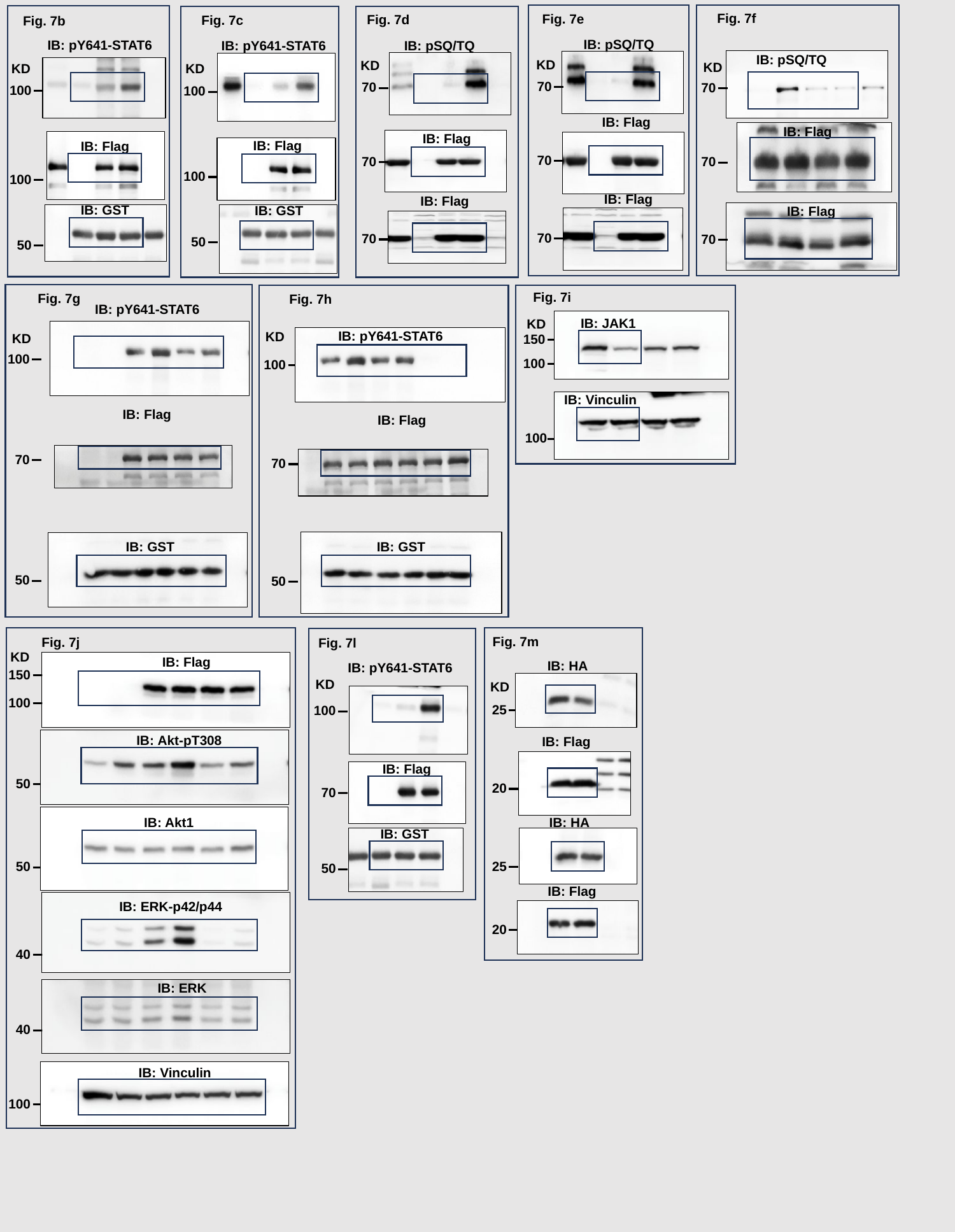

Fig. 7e
IB: pSQ/TQ
IB: Flag
IB: Flag
70
70
70
Fig. 7f
IB: pSQ/TQ
IB: Flag
IB: Flag
Fig. 7b
IB: pY641-STAT6
IB: Flag
IB: GST
Fig. 7c
IB: pY641-STAT6
IB: Flag
IB: GST
Fig. 7d
IB: pSQ/TQ
IB: Flag
IB: Flag
KD
KD
KD
KD
KD
70
70
100
100
70
70
100
100
70
70
50
50
Fig. 7i
IB: JAK1
IB: Vinculin
Fig. 7g
IB: pY641-STAT6
IB: Flag
IB: GST
Fig. 7h
IB: pY641-STAT6
IB: Flag
IB: GST
KD
KD
KD
150
100
100
100
100
70
70
50
50
Fig. 7j
IB: Flag
IB: Akt-pT308
IB: Akt1
IB: ERK-p42/p44
IB: ERK
IB: Vinculin
Fig. 7m
IB: HA
IB: Flag
IB: HA
IB: Flag
Fig. 7l
IB: pY641-STAT6
IB: Flag
IB: GST
100
70
50
KD
150
KD
KD
100
25
50
20
25
50
20
40
40
100

## Slide 8
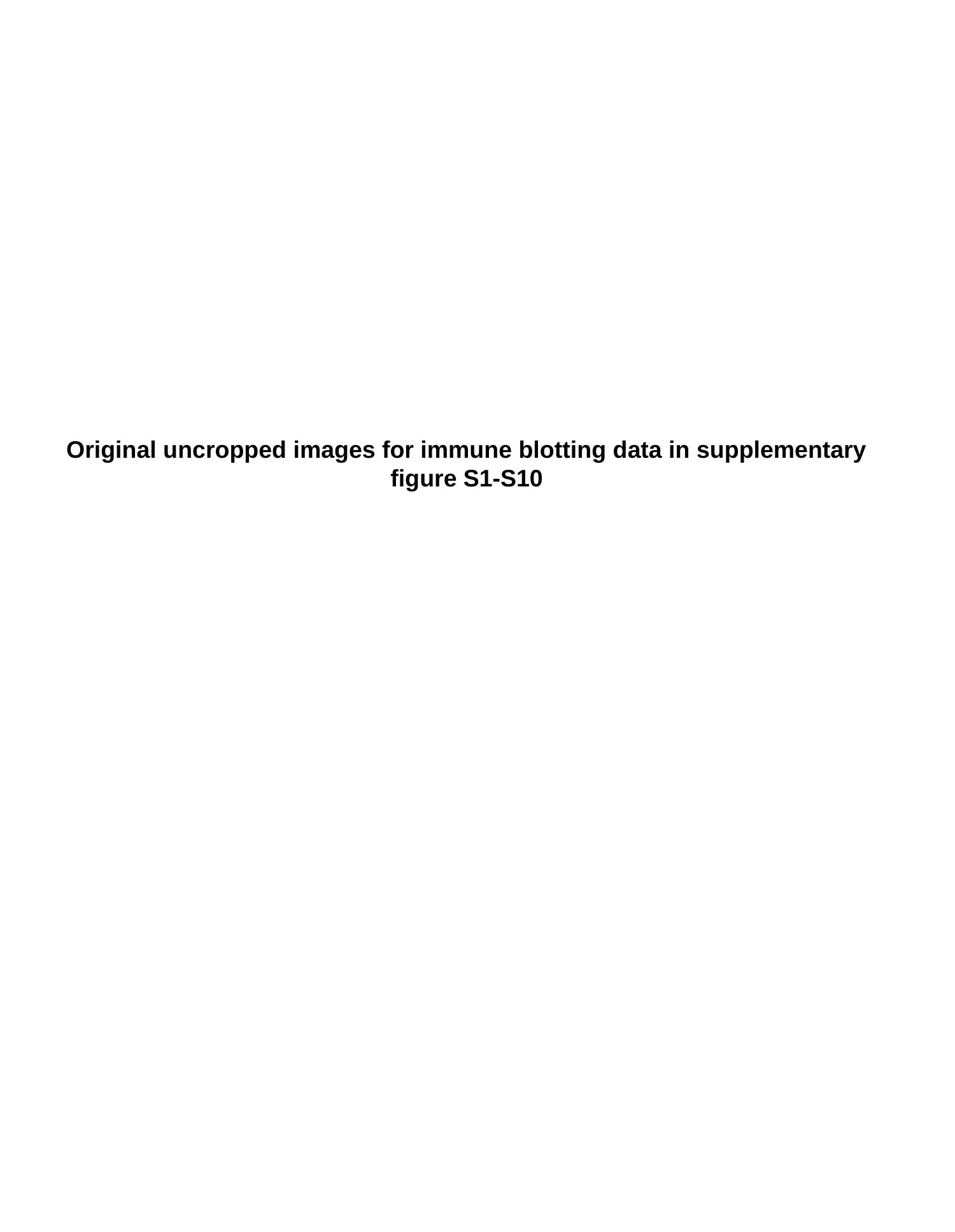

Original uncropped images for immune blotting data in supplementary figure S1-S10

## Slide 9
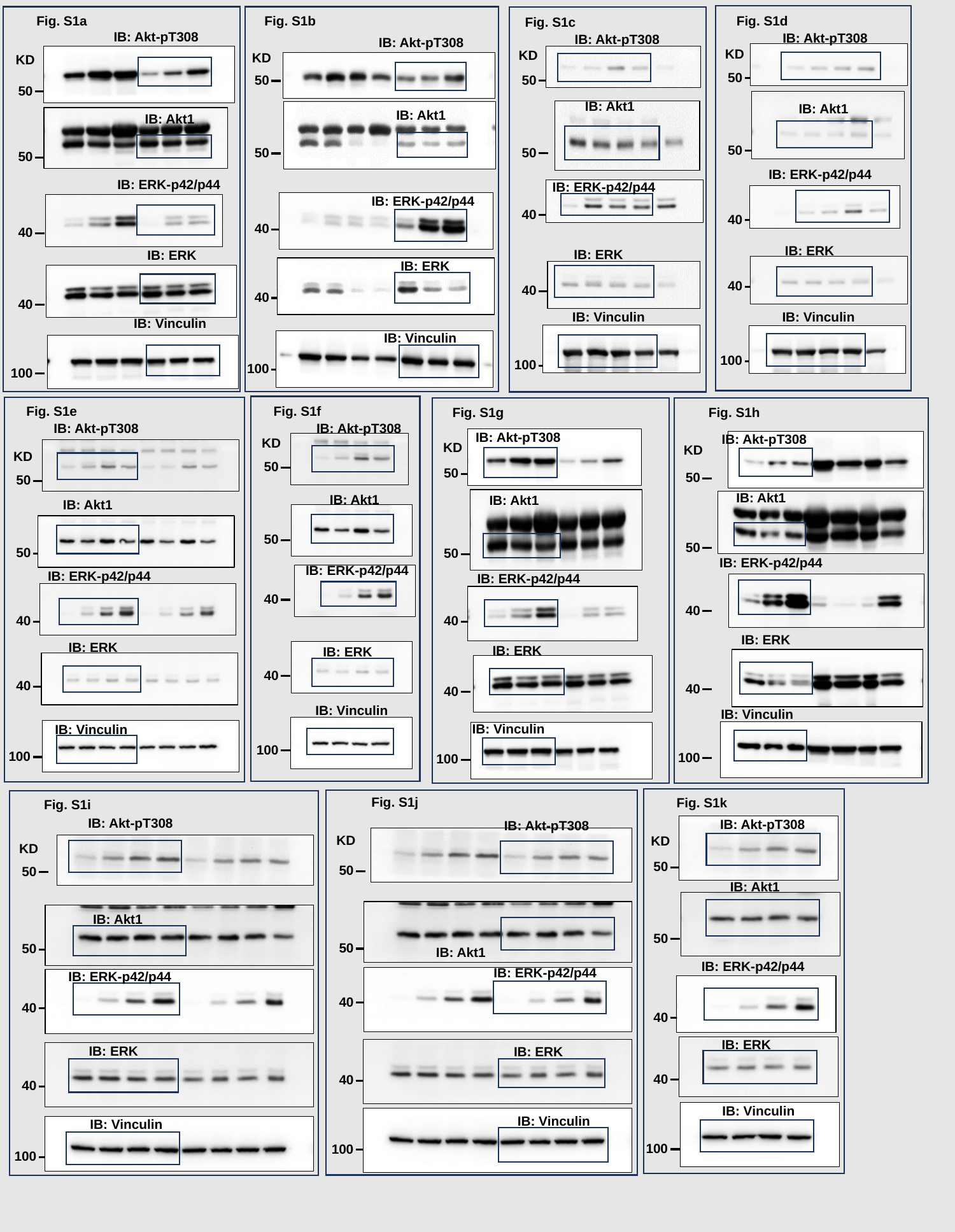

Fig. S1d
IB: Akt-pT308
KD
50
IB: Akt1
50
IB: ERK-p42/p44
40
IB: ERK
40
IB: Vinculin
100
Fig. S1a
IB: Akt-pT308
IB: Akt1
IB: ERK-p42/p44
IB: ERK
IB: Vinculin
Fig. S1b
IB: Akt-pT308
IB: Akt1
IB: ERK-p42/p44
IB: ERK
IB: Vinculin
Fig. S1c
IB: Akt-pT308
IB: Akt1
IB: ERK-p42/p44
IB: ERK
IB: Vinculin
KD
KD
KD
50
50
50
50
50
50
40
40
40
40
40
40
100
100
100
Fig. S1f
IB: Akt-pT308
IB: Akt1
IB: ERK-p42/p44
IB: ERK
IB: Vinculin
Fig. S1e
IB: Akt-pT308
IB: Akt1
IB: ERK-p42/p44
IB: ERK
IB: Vinculin
Fig. S1h
IB: Akt-pT308
IB: Akt1
IB: ERK-p42/p44
IB: ERK
IB: Vinculin
Fig. S1g
IB: Akt-pT308
IB: Akt1
IB: ERK-p42/p44
IB: ERK
IB: Vinculin
KD
KD
KD
KD
50
50
50
50
50
50
50
50
40
40
40
40
40
40
40
40
100
100
100
100
Fig. S1k
IB: Akt-pT308
IB: Akt1
IB: ERK-p42/p44
IB: ERK
IB: Vinculin
Fig. S1j
IB: Akt-pT308
IB: Akt1
IB: ERK-p42/p44
IB: ERK
IB: Vinculin
Fig. S1i
IB: Akt-pT308
IB: Akt1
IB: ERK-p42/p44
IB: ERK
IB: Vinculin
KD
KD
KD
50
50
50
50
50
50
40
40
40
40
40
40
100
100
100

## Slide 10
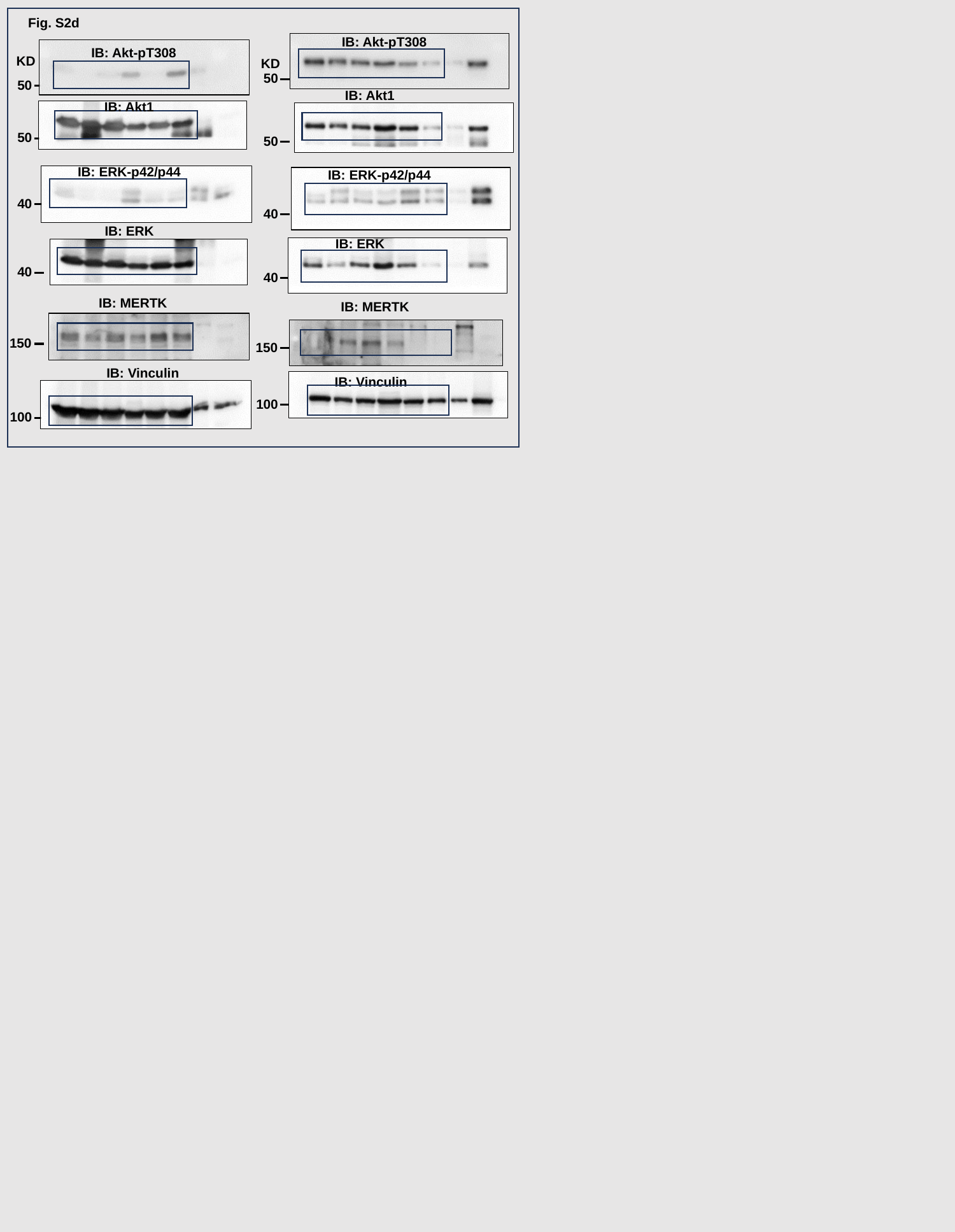

Fig. S2d
IB: Akt-pT308
IB: Akt-pT308
KD
KD
50
50
IB: Akt1
IB: Akt1
50
50
IB: ERK-p42/p44
IB: ERK-p42/p44
40
40
IB: ERK
IB: ERK
40
40
IB: MERTK
IB: MERTK
150
150
IB: Vinculin
IB: Vinculin
100
100

## Slide 11
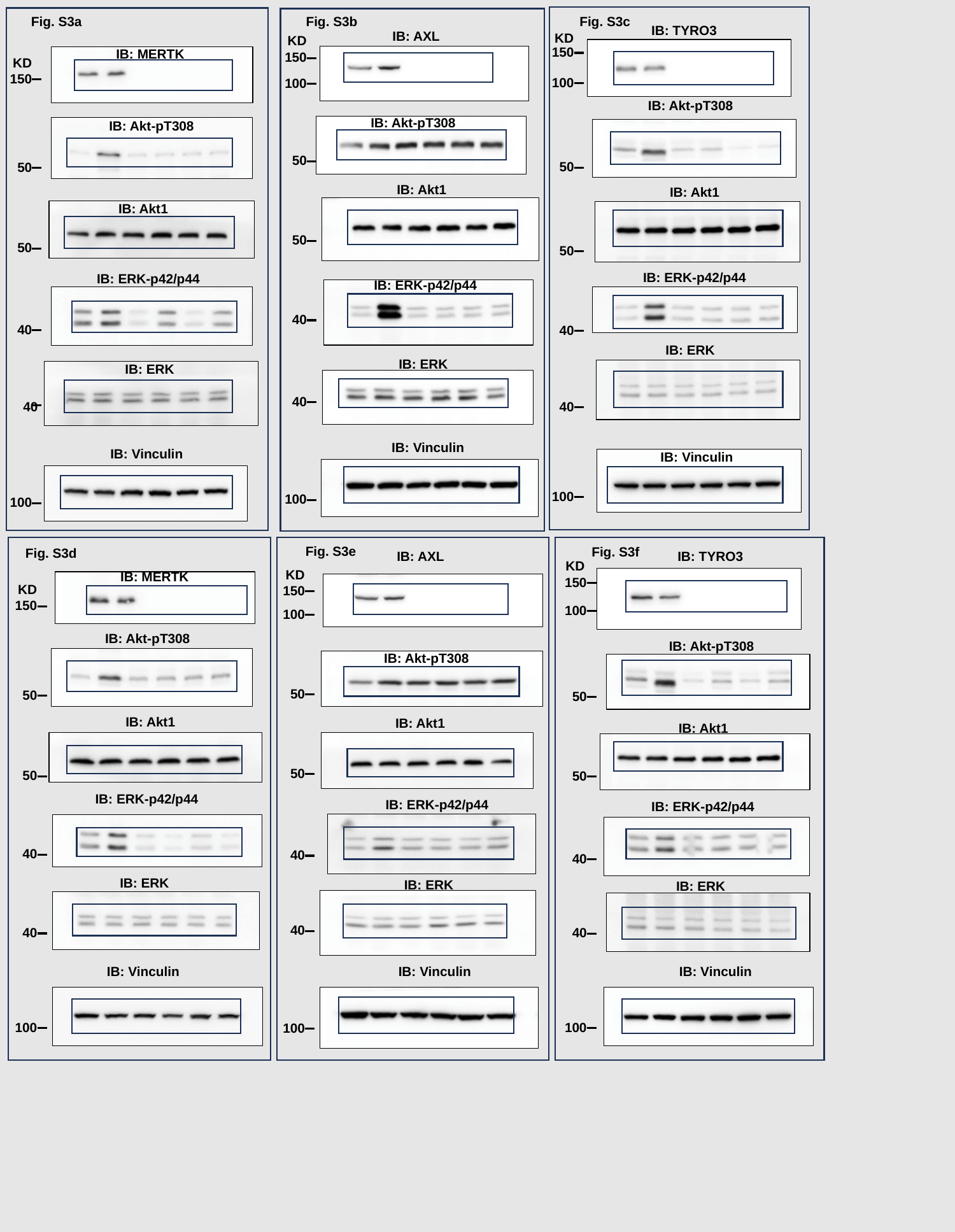

Fig. S3b
Fig. S3c
Fig. S3a
IB: TYRO3
IB: AXL
KD
KD
150
IB: MERTK
150
KD
150
100
100
IB: Akt-pT308
IB: Akt-pT308
IB: Akt-pT308
50
50
50
IB: Akt1
IB: Akt1
IB: Akt1
50
50
50
IB: ERK-p42/p44
IB: ERK-p42/p44
IB: ERK-p42/p44
40
40
40
IB: ERK
IB: ERK
IB: ERK
40
40
40
IB: Vinculin
IB: Vinculin
IB: Vinculin
100
100
100
Fig. S3e
Fig. S3f
Fig. S3d
IB: TYRO3
IB: AXL
KD
KD
IB: MERTK
150
KD
150
150
100
100
IB: Akt-pT308
IB: Akt-pT308
IB: Akt-pT308
50
50
50
IB: Akt1
IB: Akt1
IB: Akt1
50
50
50
IB: ERK-p42/p44
IB: ERK-p42/p44
IB: ERK-p42/p44
40
40
40
IB: ERK
IB: ERK
IB: ERK
40
40
40
IB: Vinculin
IB: Vinculin
IB: Vinculin
100
100
100

## Slide 12
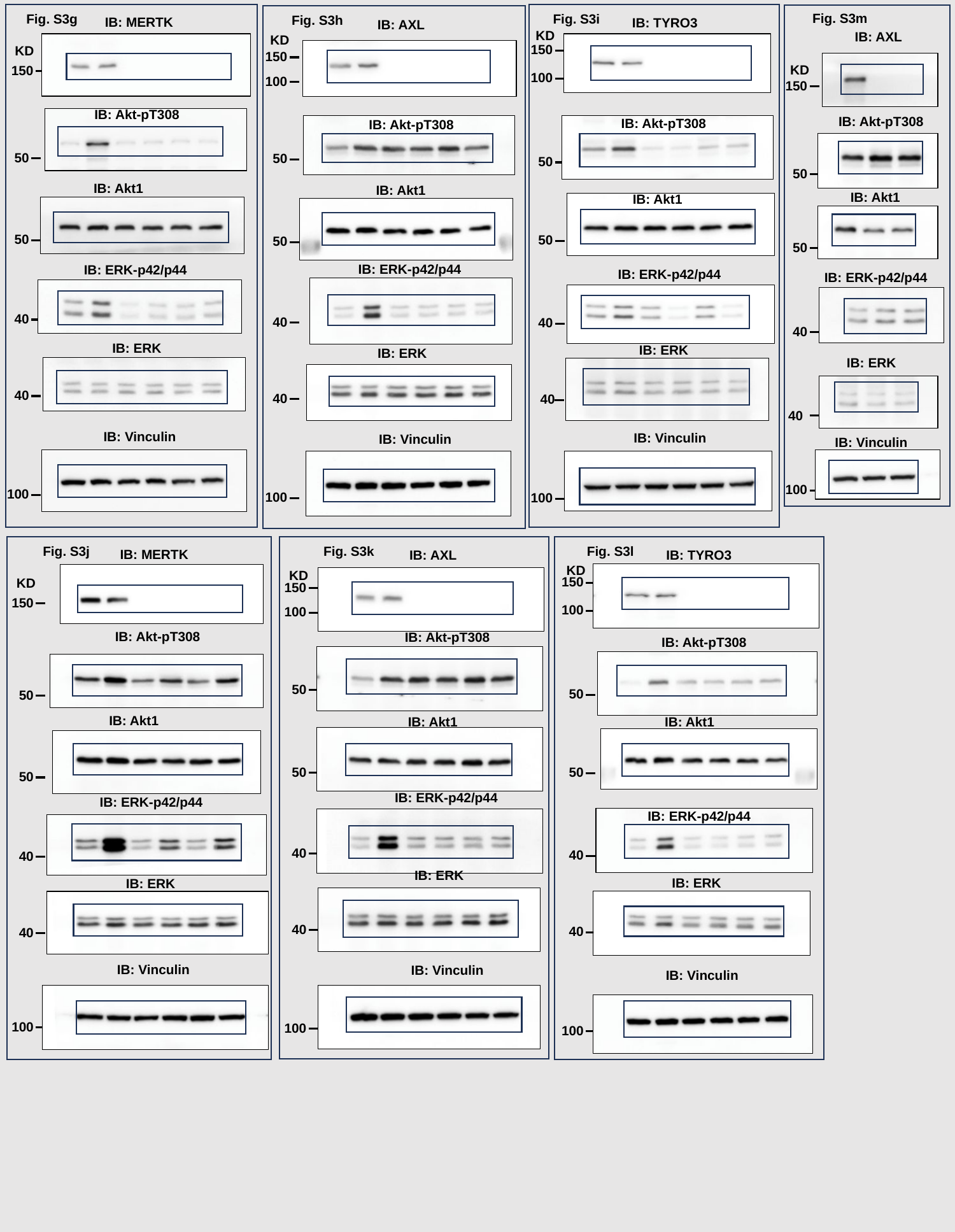

Fig. S3g
IB: MERTK
IB: Akt-pT308
IB: Akt1
IB: ERK-p42/p44
IB: ERK
IB: Vinculin
Fig. S3i
IB: TYRO3
IB: Akt-pT308
IB: Akt1
IB: ERK-p42/p44
IB: ERK
IB: Vinculin
Fig. S3m
IB: AXL
IB: Akt-pT308
IB: Akt1
IB: ERK-p42/p44
IB: ERK
IB: Vinculin
Fig. S3h
IB: AXL
IB: Akt-pT308
IB: Akt1
IB: ERK-p42/p44
IB: ERK
IB: Vinculin
KD
KD
150
KD
150
KD
150
100
100
150
50
50
50
50
50
50
50
50
40
40
40
40
40
40
40
40
100
100
100
100
Fig. S3k
IB: AXL
IB: Akt-pT308
IB: Akt1
IB: ERK-p42/p44
IB: ERK
IB: Vinculin
Fig. S3l
IB: TYRO3
IB: Akt-pT308
IB: Akt1
IB: ERK-p42/p44
IB: ERK
IB: Vinculin
Fig. S3j
IB: MERTK
IB: Akt-pT308
IB: Akt1
IB: ERK-p42/p44
IB: ERK
IB: Vinculin
KD
KD
150
KD
150
150
100
100
50
50
50
50
50
50
40
40
40
40
40
40
100
100
100

## Slide 13
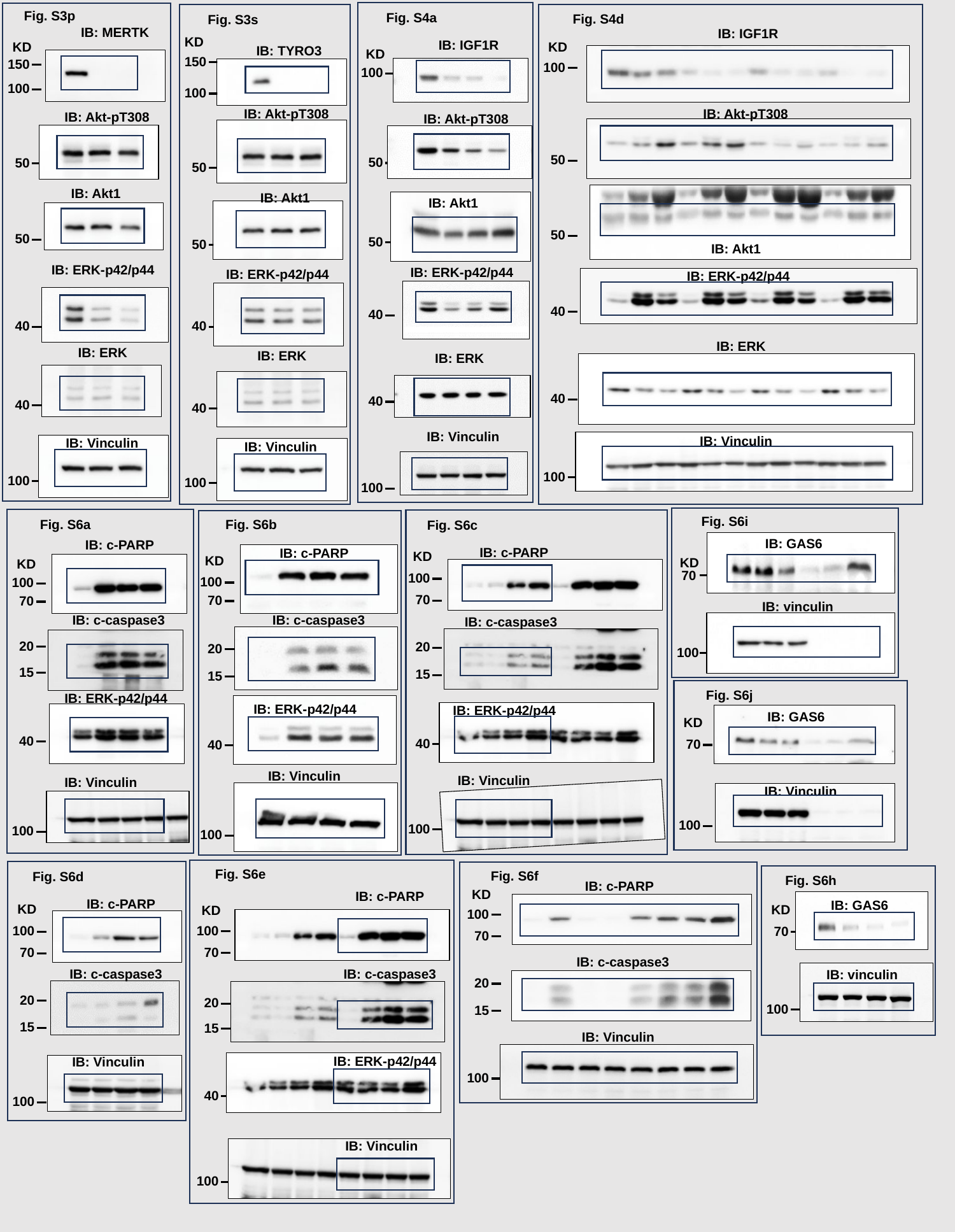

Fig. S4a
IB: IGF1R
IB: Akt-pT308
IB: Akt1
IB: ERK-p42/p44
IB: ERK
IB: Vinculin
Fig. S3p
IB: MERTK
IB: Akt-pT308
IB: Akt1
IB: ERK-p42/p44
IB: ERK
IB: Vinculin
Fig. S3s
IB: TYRO3
IB: Akt-pT308
IB: Akt1
IB: ERK-p42/p44
IB: ERK
IB: Vinculin
Fig. S4d
IB: IGF1R
IB: Akt-pT308
IB: Akt1
IB: ERK-p42/p44
IB: ERK
IB: Vinculin
KD
KD
KD
KD
150
150
100
100
100
100
50
50
50
50
50
50
50
50
40
40
40
40
40
40
40
40
100
100
100
100
Fig. S6i
IB: GAS6
IB: vinculin
Fig. S6a
IB: c-PARP
IB: c-caspase3
IB: ERK-p42/p44
IB: Vinculin
Fig. S6c
IB: c-PARP
IB: c-caspase3
IB: ERK-p42/p44
IB: Vinculin
Fig. S6b
IB: c-PARP
IB: c-caspase3
IB: ERK-p42/p44
IB: Vinculin
KD
KD
KD
KD
70
100
100
100
70
70
70
20
20
20
100
15
15
15
Fig. S6j
IB: GAS6
IB: Vinculin
KD
40
40
70
40
100
100
100
100
Fig. S6e
IB: c-PARP
IB: c-caspase3
IB: ERK-p42/p44
IB: Vinculin
Fig. S6d
IB: c-PARP
IB: c-caspase3
IB: Vinculin
Fig. S6f
IB: c-PARP
IB: c-caspase3
IB: Vinculin
Fig. S6h
IB: GAS6
IB: vinculin
KD
KD
KD
KD
100
100
100
70
70
70
70
20
20
20
100
15
15
15
100
40
100
100

## Slide 14
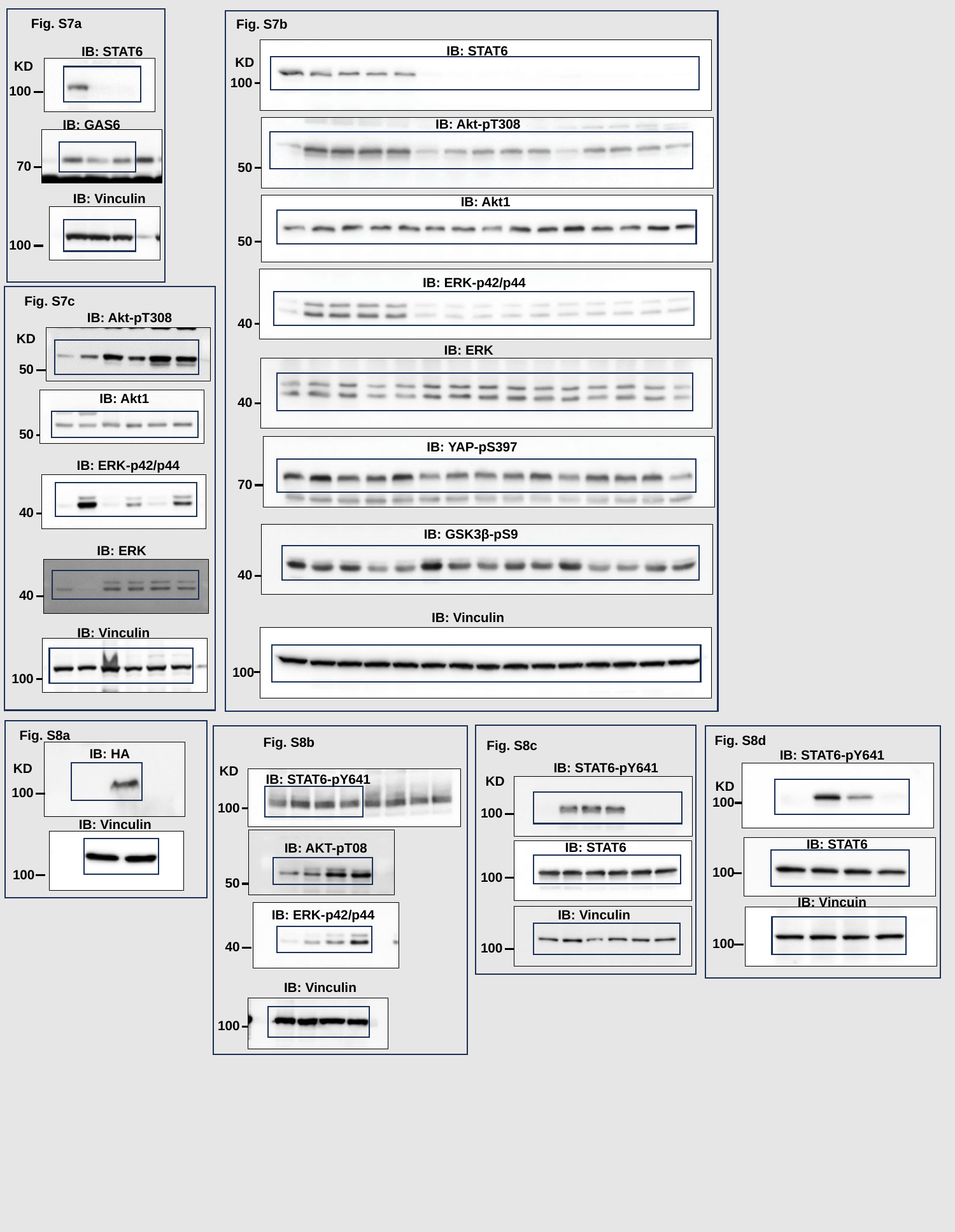

Fig. S7a
IB: STAT6
IB: GAS6
IB: Vinculin
Fig. S7b
IB: STAT6
IB: Akt-pT308
IB: Akt1
IB: ERK-p42/p44
IB: ERK
IB: YAP-pS397
IB: GSK3β-pS9
IB: Vinculin
KD
KD
100
100
70
50
50
100
Fig. S7c
IB: Akt-pT308
IB: Akt1
IB: ERK-p42/p44
IB: ERK
IB: Vinculin
40
KD
50
40
50
70
40
40
40
100
100
Fig. S8a
IB: HA
IB: Vinculin
Fig. S8c
IB: STAT6-pY641
IB: STAT6
IB: Vinculin
Fig. S8b
IB: STAT6-pY641
IB: AKT-pT08
IB: ERK-p42/p44
IB: Vinculin
Fig. S8d
IB: STAT6-pY641
IB: STAT6
IB: Vincuin
KD
KD
KD
KD
100
100
100
100
100
100
100
50
100
40
100
100

## Slide 15
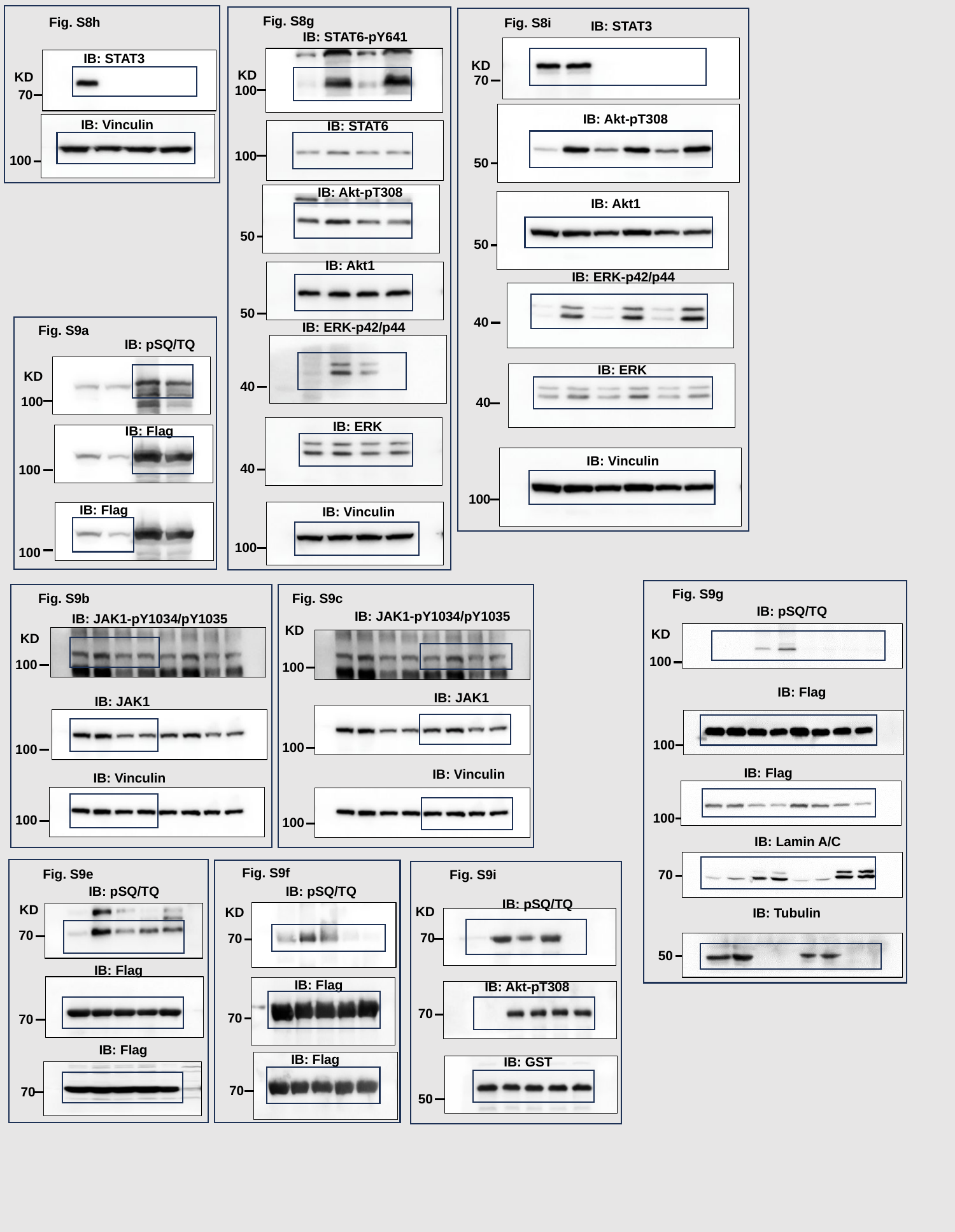

Fig. S8h
IB: STAT3
IB: Vinculin
Fig. S8g
IB: STAT6-pY641
IB: STAT6
IB: Akt-pT308
IB: Akt1
IB: ERK-p42/p44
IB: ERK
IB: Vinculin
Fig. S8i
IB: STAT3
IB: Akt-pT308
IB: Akt1
IB: ERK-p42/p44
IB: ERK
IB: Vinculin
KD
KD
KD
70
100
70
100
100
50
50
50
50
40
Fig. S9a
IB: pSQ/TQ
IB: Flag
IB: Flag
KD
40
100
40
40
100
100
100
100
Fig. S9g
IB: pSQ/TQ
KD
100
IB: Flag
100
IB: Flag
100
IB: Lamin A/C
70
IB: Tubulin
50
Fig. S9c
IB: JAK1-pY1034/pY1035
IB: JAK1
IB: Vinculin
Fig. S9b
IB: JAK1-pY1034/pY1035
IB: JAK1
IB: Vinculin
KD
KD
100
100
100
100
100
100
Fig. S9e
IB: pSQ/TQ
IB: Flag
IB: Flag
Fig. S9f
IB: pSQ/TQ
IB: Flag
IB: Flag
Fig. S9i
IB: pSQ/TQ
IB: Akt-pT308
IB: GST
KD
KD
KD
70
70
70
70
70
70
70
70
50
